# CENP-F-dependent DRP1 function regulates APC/C activity during oocyte meiosis I

**DOI:** 10.1038/s41467-022-35461-5

**Published:** 2022-12-13

**Authors:** Cheng-Jie Zhou, Xing-Yue Wang, Yan-Hua Dong, Dong-Hui Wang, Zhe Han, Xiao-Jie Zhang, Qing-Yuan Sun, John Carroll, Cheng-Guang Liang

**Affiliations:** 1 State Key Laboratory of Reproductive Regulation & Breeding of Grassland Livestock, School of Life Sciences, Inner Mongolia University, 010070 Hohhot, Inner Mongolia China; 2 Fertility Preservation Lab, Guangdong-Hong Kong Metabolism & Reproduction Joint Laboratory, Reproductive Medicine Center, Guangdong Second Provincial General Hospital, 510317 Guangzhou, China; 3 Development and Stem Cells Program, Department of Anatomy and Developmental Biology, Monash Biomedicine Discovery Institute, Monash University, Melbourne, VIC 3800 Australia

**Keywords:** Chromosome segregation, Checkpoint signalling, Oogenesis

## Abstract

Chromosome segregation is initiated by cohesin degradation, which is driven by anaphase-promoting complex/cyclosome (APC/C). Chromosome cohesin is removed by activated separase, with the degradation of securin and cyclinB1. Dynamin-related protein 1 (DRP1), a component of the mitochondrial fission machinery, is related to cyclin dynamics in mitosis progression. Here, we show that DRP1 is recruited to the kinetochore by centromeric Centromere protein F (CENP-F) after nuclear envelope breakdown in mouse oocytes. Loss of DRP1 during prometaphase leads to premature cohesin degradation and chromosome segregation. Importantly, acute DRP1 depletion activates separase by initiating cyclinB1 and securin degradation during the metaphase-to-anaphase transition. Finally, we demonstrate that DRP1 is bound to APC2 to restrain the E3 ligase activity of APC/C. In conclusion, DRP1 is a CENP-F-dependent atypical spindle assembly checkpoint (SAC) protein that modulates metaphase-to-anaphase transition by controlling APC/C activity during meiosis I in oocytes.

## Abstract (web summary)

Spindle assembly checkpoint controls anaphase onset and guarantees appropriate chromosome segregation. Here, the authors report that dynamin-related protein 1 is recruited to kinetochores by CENP-F to regulate metaphase-to-anaphase transition by controlling APC/C activity in mouse oocyte meiosis

## Introduction

During mammalian meiosis, the fully grown germinal vesicle (GV) stage oocyte undergoes nuclear envelope breakdown (NEBD), followed by chromosome condensation and spindle assembly. Subsequently, the spindle microtubules capture chromosomes at the equatorial plate at metaphase I (MI) and prepare for chromosome segregation^1^. To guarantee correct chromosome segregation, the spindle assembly checkpoint (SAC) controls anaphase onset until all kinetochores are attached to the spindle microtubules and homologous chromosomes are accurately aligned to the metaphase plate^2,3^. Once the SAC is satisfied, anaphase is initiated, and the chromosomes separate^4^. The oocyte then extrudes the first polar body (PB1) and rearrests at metaphase II (MII) until fertilisation. The effector of the SAC is the mitotic checkpoint complex (MCC), which includes mitotic arrest deficient-like 2 (MAD2), budding uninhibited by benzimidazoles 3 (BUB3), bub-related 1 (BUBR1), and cell-division cycle protein 20 homologue (CDC20). MCC-mediated sequestration of CDC20, the mitotic activator of the anaphase-promoting complex/cyclosome (APC/C)^5–9^ during prometaphase of mitosis, inhibits the APC/C^6^. In oocyte meiosis, a prerequisite for anaphase onset is the release of MCC from the kinetochore^10^, which frees CDC20 to activate the APC/C. Activation of the APC/C drives the polyubiquitylation and degradation of cyclinB1 and securin. Degradation of these substrates activates separase, which removes cohesin from homologous chromosomes (in meiosis I) or sister chromatids (in meiosis II), allowing for chromosome separation^11^.

Centromere protein F (CENP-F, mitosin), a component of the outer kinetochore, is the key regulator of kinetochore-microtubule (K-MT) attachment^12,13^ and is indispensable for spindle assembly in mitosis^13,14^. In somatic cells, loss of CENP-F labilises K-MT attachment^15^, which correlates with chromosome misalignment in chromosome alignment-maintaining phosphoprotein (CAMP) mutants^16^, suggesting that CENP-F depletion activates the SAC. Whereas CENP-F loss reportedly weakens centromeric cohesion and leads to premature separation of sister chromatids^13^. Moreover, depletion of CENP-F is described to cause defective localisation of CENP-E^17,18^, BUBR1, and MAD1^19^ at kinetochores, indicating SAC inactivation. These conflicting reports illustrate that the inter-relationship between CENP-F and SAC activation remains unclear.

Here, we report a CENP-F effector protein, dynamin-related protein 1 (DRP1), which is recruited to kinetochores by CENP-F to regulate SAC activation during the metaphase-to-anaphase transition. DRP1 is conserved among mammals, *C. elegans*, and *Drosophila*, and is an essential mitochondrial fission mediator. In addition, DRP1 targets microtubules^20^ to regulate spindle assembly^21^ and chromosome segregation^22^, which have been attributed to the mitochondrial functions of DRP1. In rodents, DRP1 knockout in primary oocytes disturbs endoplasmic reticulum, secretory vesicle distribution, and oocyte meiotic resumption^23^. Loss of DRP1 in fully grown oocytes leads to spindle migration failure^24^. Moreover, DRP1 knockout in murine embryos leads to brain hypoplasia and death after embryonic day 12.5^25^. As expected, the role of DRP1 in these cells was related to its function in mitochondrial fission. Interestingly, DRP1 is also associated with the dynamics of cyclins and cyclin-dependent kinases (CDKs), including cyclinB^22^, cyclinC^26,27^, cyclinE^22,28^, CDK1, and CDK5^20,29^, in mammalian somatic cells. We hypothesise that DRP1 controls metaphase-to-anaphase transition in oocytes via an alternative mechanism of direct regulation of APC/C. Our study suggests DRP1 is a CENP-F-dependent, atypical MCC member that mediates metaphase-to-anaphase transition by driving APC/C activity in mouse oocyte meiosis. These findings extend the role of DRP1 as a cell cycle regulator functions during oocyte meiosis maturation.

## Results

### CENP-F stabilises arm cohesion to prevent chromosomes premature segregation during meiosis I

**Fig. 1:**
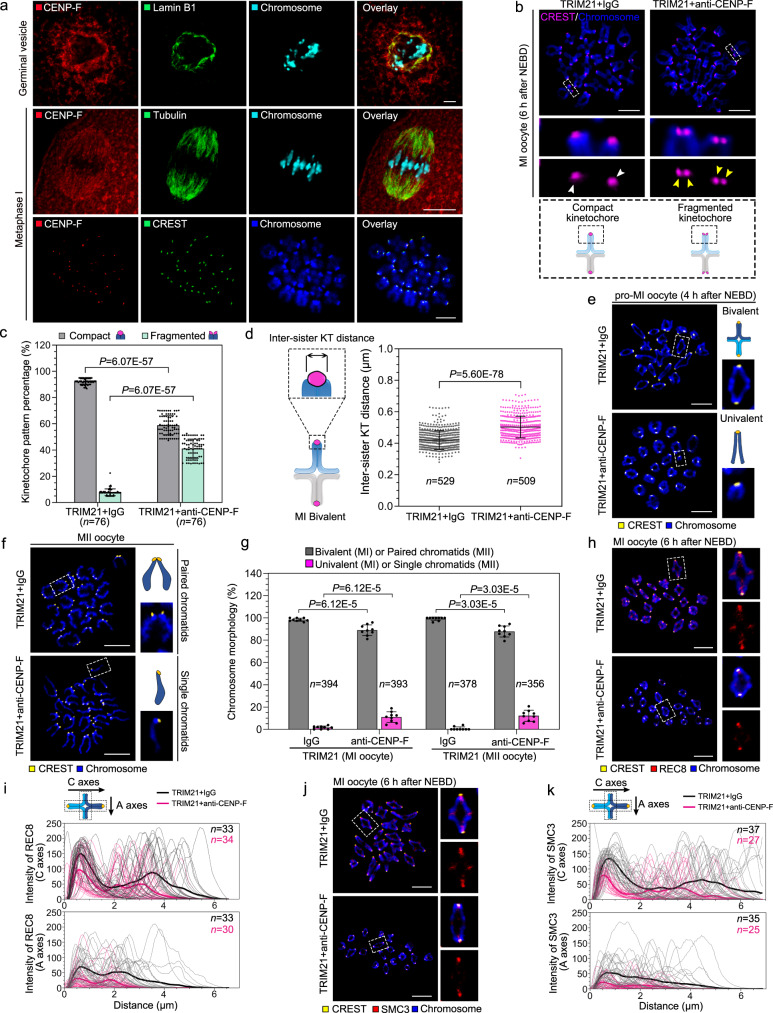
CENP-F is required for the protection of chromosome cohesion during oocyte meiosis I. **a** CENP-F localisation (red) during mouse oocyte meiosis. Antibodies against Lamin B1 (row 1, green) and Tubulin (row 2, green) were used to mark the nuclear envelope and spindle, respectively. **b**–**d** Kinetochore configurations (**b**), percentages (**c**), and inter-sister kinetochore (KT) distance (**d**) in control or CENP-F-Trim MI stage oocytes. Enlarged images in (**b**) show representative compact (white arrows) or fragmented (yellow arrows) kinetochores. Five independent replicates were performed for (**c**, **d**). **e**–**g** Chromosome configurations of pro-MI (**e**) and MII (**f**) oocyte and their occurrence after CENP-F Trim-Away (**g**). Enlarged images show representative bivalent or univalent chromosomes (**e**) or paired or single chromatids (**f**). Nine independent replicates were performed for (**g**). **h** MI stage oocytes were stained for REC8 (red) after CENP-F Trim-Away. Enlarged images show representative results. **i** Fluorescence intensity of REC8 on centromeric arms (*C* axes) and acentric arms (*A* axes) in (**h**) was measured. Three independent replicates were performed. **j** MI stage oocytes were stained for SMC3 (red) after CENP-F Trim-Away. Enlarged images show representative results. **k** Fluorescence intensity of SMC3 on centromeric arms (*C* axes) and acentric arms (*A* axes) in (**j**) was measured. Three independent replicates were performed. Chromosomes were stained with Hoechst 33342 (blue) in (**a**, **b**, **e**, **f**, **h**, **j**), and kinetochores were marked with CREST (green in (**a**), row 3, magenta in (**b**), and yellow in (**e**, **f**, **h**, **j**). Data are presented as the mean ± standard deviation (S.D.). *P* values were calculated based on nonparametric Kruskal–Wallis tests for (**c**, **g**), or unpaired Student’s *t* test (two-tailed) for (**d**). Black arrows in (**i**, **k**) show the measurement direction. *n* in graphs refers to the total number of oocytes in (**c**, **g**), kinetochores in (**d**), or chromosomes in (**i**, **k**), respectively. The white dotted frames in (**b**, **e**, **f**, **h**, **j**) indicate the region shown in detail. Scale bar, 5 μm. NEBD, nuclear envelope breakdown; MI, metaphase I; MII, metaphase II. Representative stainings from at least three independent repeats are shown. Source data are provided as a Source Data file. See also in Supplementary Fig. 1.

CENP-F is a multifunctional microtubule-binding protein that undergoes dynamic changes in distribution during mitosis^30^. In the mouse oocyte, CENP-F showed a similar dynamic pattern of distribution, localising to nuclear membranes at the GV stage and to spindle microtubules at the examined prophase I, metaphase I, anaphase I, telophase I, and metaphase II. During these stages of meiotic progression, CENP-F was also observed in a punctate pattern in the cytoplasm. Remarkably, CENP-F immunofluorescence decorated kinetochores at metaphase I and metaphase II and was also found at the spindle mid-zone at telophase I (Fig. 1a and Supplementary Fig. 1a).

To investigate the role of CENP-F in oocyte meiosis and particularly its role at kinetochores, we used ‘Trim-Away’, a highly effective method for rapidly and specifically degrading target proteins^31–33^. Trim-Away using an anti-CENP-F antibody, removed approximately 85% of CENP-F protein within 2 h as determined by western blot analysis (Supplementary Fig. 1b) and immunofluorescence (Supplementary Fig. 1c). To examine the effect of CENP-F depletion on kinetochores, we labelled chromosome spreads with the kinetochore-specific marker CREST^34^. We found that kinetochores of CENP-F-depleted MI stage oocytes were split into two lobes (Fig. 1b, c). In addition, the inter-kinetochore distance between sister chromatids increased significantly in CENP-F-depleted oocytes compared to the controls (Fig. 1d). These results indicate that CENP-F is necessary to maintain the association between sister chromatids. This was further demonstrated by the observation that approximately 11% of CENP-F-depleted oocytes underwent premature bivalent segregation in meiosis I (Fig. 1e, g), and approximately 12% of CENP-F-depleted oocytes underwent sister chromatid segregation at the MII stage (Fig. 1f, g).

The protein ring complex cohesion maintains the tight association of homologues and sister chromatids. The separation of homologues or sister chromatids at anaphase I or II, respectively, is only triggered when cohesin is destroyed by separase^35^. We, therefore, evaluated the presence of cohesion after the depletion of CENP-F by measuring the presence of the cohesin subunits, REC8 and SMC3, on the centromeric (*C* axes) and acentric axes (*A* axes) of the homologues. Although centromeric cohesin appeared to be primarily protected, REC8 and SMC3 were effectively lost from the chromosome arms after CENP-F depletion (Fig. 1h–k). Taken together, these data suggest that CENP-F is essential for stabilising cohesin on MI chromosomes.

### CENP-F recruits DRP1 to kinetochores during meiosis

**Fig. 2:**
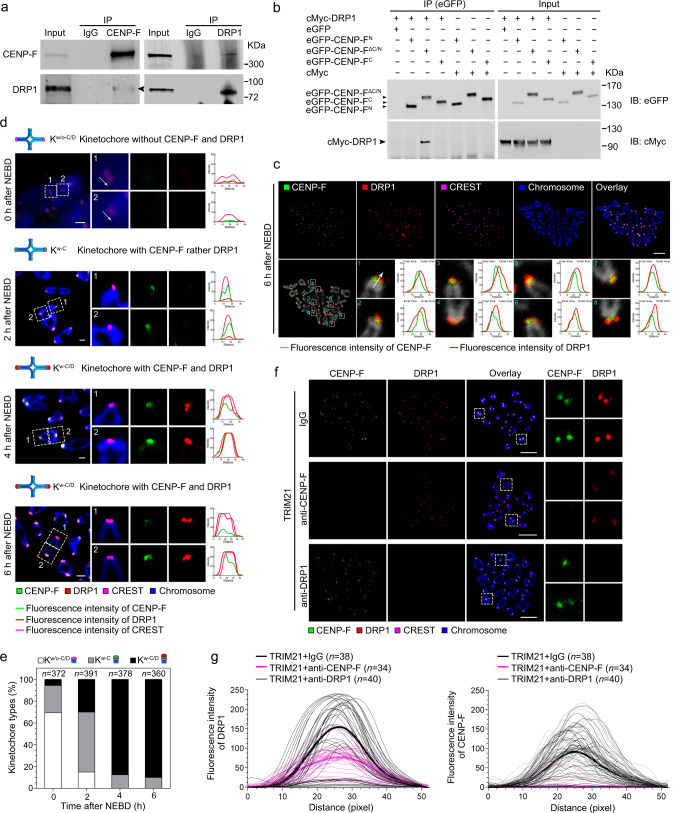
CENP-F recruits DRP1 to the kinetochores during oocyte meiosis I. **a** Co-IP was performed to prove the interaction between CENP-F and DRP1. Per lysate containing 1000 oocytes was incubated with anti-CENP-F and anti-DRP1, respectively. IP eluates were used for immunoblot with anti-CENP-F and anti-DRP1, respectively. The black arrow shows the band of DRP1. **b** The expression plasmids of PCS2 + -*eGFP*-*Cenp-f*^C^, PCS2 + -*eGFP*-*Cenp-f*^ΔC/N^ or PCS2 + -*eGFP*-*Cenp-f*^ΔN^ were co-transfected with PCS2 + -*cMyc*-*Drp1* to HEK-293 cells for 48 h, respectively. The lysates were incubated with anti-eGFP. IP eluates were used for immunoblot with anti-eGFP and anti-cMyc, respectively. One-tenth of the input cell lysates were used for immunoblot. **c** Co-localisation of CENP-F and DRP1 during meiosis I. Enlarged images show representative results. White arrows indicate the measurement direction of fluorescence intensity from the inner to the outer kinetochore. The distance is shown in pixel. Scale bar, 5 μm. **d**, **e** Frequency of CENP-F and DRP1 recruited to the kinetochores during meiosis I. White arrows show the measurement direction of the fluorescence intensity. If not defined, the measurement direction starts from the left of the kinetochore to the right in the enlarged images. Fluorescence intensities of CENP-F (green line), DRP1 (red line) and CREST (magenta line) are shown in line graph. The distance shows in pixel. Scale bar, 1 μm. K, kinetochores; C, CENP-F; D, DRP1. **f** CENP-F and DRP1 localisation on kinetochores after CENP-F or DRP1 Trim-Away. The amplified four images in row 1, row 2 and row 3 show CENP-F and DRP1 co-localised on kinetochores, CENP-F loss reduced DRP1 localisation, and unaffected CENP-F localisation after DRP1 loss, respectively. Scale bar, 5 μm. **g** Fluorescence intensities of DRP1 and CENPF in (**f**) were measured. The distance is shown in pixel. Chromosomes were stained with Hoechst 33342 (blue), and kinetochores were marked with CREST (magenta in **c**, **d**, **f**). *n* in the graphs refers to the number of kinetochores in (**e**, **g**). The white dotted frames in (**c**, **d**, **f**) indicate the region shown in detail. Three independent replicates were performed for (**e**, **g**). NEBD, nuclear envelope breakdown. Representative blots or stainings from at least three independent repeats are shown. Source data are provided as a Source Data file. See also in Supplementary Figs. 2–4.

To further investigate how CENP-F stabilises cohesin, we collected 1000 GV, MI, and MII stage oocytes for CENP-F immunoprecipitation. Eluates were prepared for SDS-PAGE, followed by silver staining. Target bands common to each stage were collected and analysed with MALDI/MS to identify CENP-F interacting proteins (Supplementary Fig. 2a). The target proteins of the GV (L1-L4), MI (L1′-L4′), and MII (L1″-L4″) groups were confirmed with the top hit being DRP1 (Supplementary Fig. 2b–d). Furthermore, co-immunoprecipitation (Co-IP) showed that anti-CENP-F co-precipitated DRP1, and anti-DRP1 co-precipitated CENP-F (Fig. 2a). These observations suggest that CENP-F and DRP1 may interact in oocytes, although the nature of this interaction remains unclear.

CENP-F contains a coiled-coil domain on its N-terminal end, tandem repeats internally, and leucine zippers and structural maintenance of chromosomes (SMC) domains on its C-terminal end^36^. To define which region of CENP-F interacts with DRP1, three fragments of the depletion cloning construct were selected, including *Cenp-f*^ N^ (1-963), *Cenp-f*^ ΔC/N^ (964-1983), and *Cenp-f*^C^ (1984-2999), and fused with the *eGFP* sequence. *Drp1* was fused with *cMyc* (Supplementary Fig. 2e) and then co-transfected with each of the *Cenp-f* constructs into HEK-293 cells for Co-IP. We found that the internal fragment, CENP-F^ΔC/N^, was able to bind DRP1 (Fig. 2b).

Given the evidence that CENP-F and DRP1 interact, we next investigated whether they co-localise at the centromere. We began by confirming the co-localisation of DRP1 and mitochondria^37^. Our results showed that exogenous cMyc-tagged DRP1 co-localised with mitochondria in GV and MI stage oocytes (Supplementary Fig. 3a). Next, we expressed exogenous cMyc-tagged DRP1 in meiosis I oocytes and performed immunostaining for cMyc on chromosome spreads. cMyc was detected at centromeres as evidenced by co-localisation with the constitutive centromeric protein CREST (Supplementary Fig. 3b). Centromeric localisation was further confirmed using three different anti-DRP1 antibodies (Supplementary Fig. 3c–e). Moreover, DRP1 was localised at centromeres at the end of microtubules (Supplementary Fig. 3f), and there was some evidence of punctate microtubule labelling. Co-localisation analysis on chromosome spreads showed that CENP-F and DRP1 signals partially overlapped, with DRP1 appearing just distal to CENP-F on the kinetochore (Fig. 2c).

To investigate the dynamics of CENP-F and DRP1, we examined their localisation at the kinetochore during meiosis I. Kinetochores were categorised by the absence of both CENP-F and DRP1 (K^w/o-C/D^), the presence of CENP-F but not DRP1 (K^w-C^), or the presence of both proteins (K^w-C/D^). Fluorescence intensities of CENP-F, DRP1, and CREST were quantified in line graphs (Fig. 2d), and kinetochores of each type were quantified (Fig. 2e). Just after NEBD, the majority of oocytes showed no evidence of labelling for either protein at the kinetochore (K^w/o-C/D^), whereas about 20% showed evidence of CENP-F alone, and only 5% showed evidence of both proteins being present (Fig. 2d panel 1, 2e, and Supplementary Fig. 4a). Two hours after NEBD, around 85% of kinetochores had recruited CENP-F (K^w-C^), and approximately 35% of these also showed evidence of DRP1 (K^w-C/D^) (Fig. 2d panel 2, 2e, and Supplementary Fig. 4b). By 4 and 6 h after NEBD, all kinetochores showed evidence of CENP-F, with around 10% showing no evidence of DRP1 (K^w-C^) (Fig. 2d panels 3-4, 2e, and Supplementary Fig. 4c, d). Interestingly, the recruitment of DRP1 without CENP-F was never observed.

These observations demonstrate that CENP-F localises to kinetochores during chromosome alignment and recruits DRP1. To test this further, we depleted CENP-F using the Trim-Away approach and monitored DRP1 localisation and vice-versa. We first confirmed the high efficiency of DRP1 knockdown with the Trim-Away approach (Supplementary Fig. 3g). Consistent with our hypothesis, CENP-F depletion abolished the recruitment of DRP1, whereas depletion of DRP1 did not affect centromeric CENP-F localisation (Fig. 2f, g). These data suggest that centromeric CENP-F directly recruits DRP1 to the kinetochore during meiosis.

### DRP1 depletion phenocopies the effect of CENP-F depletion on kinetochore organisation

**Fig. 3:**
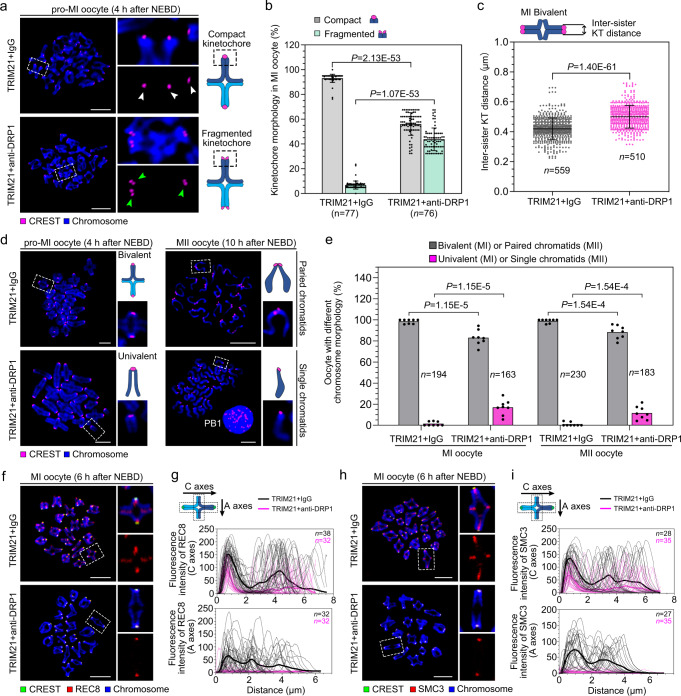
DRP1 loss leads to SAC defects and premature chromosome separation. **a** Kinetochore configurations in control or DRP1 null oocytes. White arrows indicate the compact kinetochores, and green arrows indicate the fragmented kinetochores. **b** The occurrence frequency of kinetochore morphology in (**a**) in three independent replicates. **c** Measurement of inter-sister kinetochore distance of MI bivalents in (**a**). Three independent replicates were performed. **d** Chromosome configurations of control or DRP1 null oocytes at MI and MII stages. Enlarged images show representative chromosomes. **e** The occurrence frequency of chromosome morphology in (**d**) in eight independent replicates. **f** MI stage oocyte microinjected with TRIM21 + IgG or TRIM21 + anti-DRP1 were stained for REC8 (red). Enlarged images show representative results. **g** Fluorescence intensity of REC8 on centromeric arms (*C* axes) and acentric arms (*A* axes) in (**f**) was measured, respectively. Three independent replicates were performed. **h** MI stage oocytes microinjected with TRIM21 + IgG or TRIM21 + anti-DRP1 were stained for SMC3 (red). Enlarged images show representative results. **i** Fluorescence intensity of SMC3 on centromeric arms (*C* axes) and acentric arms (*A* axes) in (**h**) was measured, respectively. Three independent replicates were performed. Chromosomes were stained with Hoechst 33342 (blue), and kinetochores were marked with CREST (magenta in **a**, **d**, and green in **f**, **h**). Data are presented as the mean ± standard deviation (S.D.). *P* values were calculated based on nonparametric Kruskal–Wallis tests for (**b**, **e**), and unpaired Student’s *t* test (two-tailed) for (**c**). Black arrows in (**g**, **i**) show the measurement direction. *n* in graphs refers to the number of oocytes in (**b**, **e**), kinetochores in (**c**) and chromosomes in (**g**, **i**), respectively. The white dotted frames in (**a**, **d**, **f** and **h**) indicate the region shown in detail. Scale bar, 5 μm. NEBD, nuclear envelope breakdown; MI, metaphase I; MII, metaphase II. Representative stainings from at least three independent repeats are shown. Source data are provided as a Source Data file.

Given that chromosome spreads in CENP-F-depleted oocytes showed a decrease in cohesion (Fig. 1h–k) and our hypothesis that the interaction of CENP-F and DRP1 is essential for maintaining cohesion, we expected loss of DRP1 to phenocopy loss of CENP-F. We used Trim-Away-mediated degradation of DRP1 to test this hypothesis. Analysis of chromosome spreads revealed that kinetochores of DRP1-depleted oocytes showed many of the same features observed in CENP-F-depleted oocytes. Specifically, kinetochores were fragmented into two lobes (Fig. 3a, b), inter-sister kinetochore distance was increased (Fig. 3c), and premature chromosome segregation in MI and MII stage oocytes was more frequent (Fig. 3d, e). Additionally, REC8 and SMC3 were reduced on the centromeric (*C* axes) and acentric axes (*A* axes) of chromosome arms, whereas centromeric cohesin remained intact (Fig. 3f–i). Thus, DRP1 at kinetochores may be crucial for protecting cohesin during MI.

### DRP1 regulates the timing of anaphase I onset

**Fig. 4:**
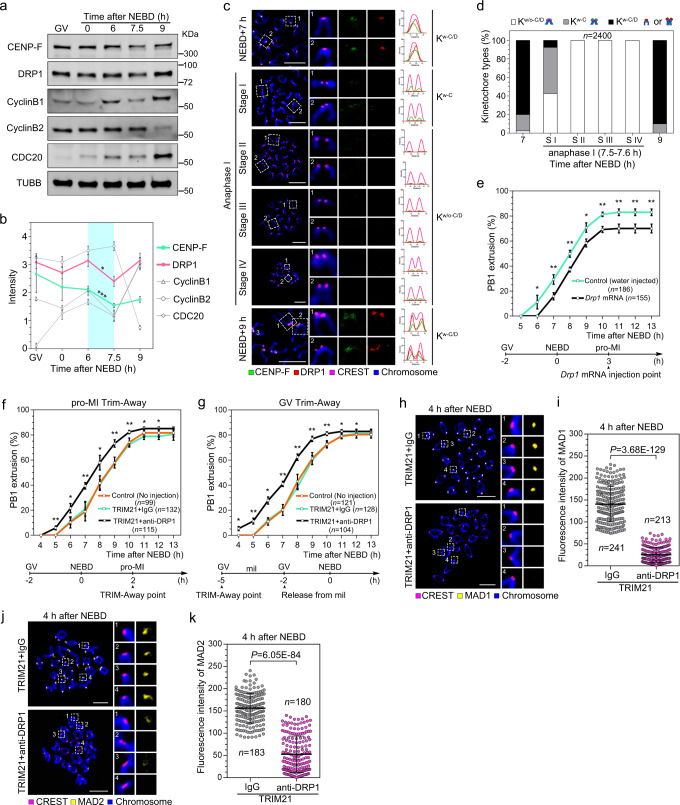
DRP1 degradation is required for metaphase I to anaphase I transition. **a**, **b** Expression (**a**) and intensities (**b**) of cell cycle related proteins during oocyte meiosis. β-Tubulin was used as the loading control. **c** Frequency of CENP-F and DRP1 disaggregation from the kinetochores after NEBD. Stages I–IV of anaphase were determined by the distance of homologous chromosomes after chromosome spreading. Fluorescence intensities of CENP-F (green line), DRP1 (red line) and CREST (magenta line) are shown in the line graph. Measurement direction starts from the left of the kinetochore to the right in the enlarged images. The distance is shown in pixel. **d** The occurrence of kinetochore types in (**c**) from three independent replicates. **e** PB1 extrusion rates in control and *Drp1* mRNA injected oocytes at consecutive time points after NEBD. *Drp1* mRNA was injected into oocytes 3 h after NEBD. Three independent replicates were performed. **f**, **g** PB1 extrusion rates in control, TRIM21 + IgG and TRIM21 + anti-DRP1 oocytes at consecutive time points after NEBD. The injection was performed at 2 h after NEBD (**f**) or at the GV stage (**g**). Three independent replicates were performed. **h**–**k** Localisation and fluorescence intensity of MAD1 (**h**, **i**) and MAD2 (**j**, **k**) after DRP1-Trim-Away. Enlarged images show representative results. Three independent replicates were performed for (**i**, **k**). Chromosomes were stained with Hoechst 33342 (blue), and kinetochores were marked with CREST (magenta). Data are presented as the mean ± standard deviation (S.D.). *P* values were calculated based on nonparametric Kruskal–Wallis tests for (**e**, **f** and **g**), or unpaired Student’s *t* test (two-tailed) for (**b**, **i** and **k**). *P < 0.05, **P < 0.01, and ***P < 0.001. *n* in graphs refers to the total number of kinetochores in (**d**, **i** and **k**) or oocytes in (**e**, **f** and **g**), respectively. The white dotted frames in (**c**, **h**, and **j**) indicate the region shown in detail. Scale bar: 5 μm. NEBD, nuclear envelope breakdown; PB1, first polar body. Representative blots or stainings from at least three independent repeats are shown. The exact *P* values for (**b**, **e**–**g**) are provided as a Source Data file. See also in Supplementary Figs. 5–7.

To investigate the role of DRP1 during meiosis, we examined the relative expression levels of DRP1 and CENP-F during meiotic progression by western blot. Interestingly, both proteins showed a decreasing trend during the metaphase-to-anaphase transition, similar to the APC/C substrates, cyclinB1 and CDC20 (Fig. 4a, b). To examine these dynamics at the single oocyte level, we performed immunofluorescence for CENP-F and DRP1 on chromosome spreads from anaphase through MII (7–9 h after NEBD). We found that centromeric DRP1 began to degrade after metaphase I, with 80% of kinetochores staining positive for DRP1 7 h after NEBD (Fig. 4c, d and Supplementary Fig. 5a), whereas virtually all kinetochores were positive for CENP-F, as expected. At the onset of anaphase I (stage I), DRP1 labelling was evident on only 7.5% of kinetochores, whereas 50% remained CENP-F positive. By the later stages of anaphase (stage II, III, and IV), kinetochores were completely bereft of DRP1 and CENP-F, and labelling was restored at metaphase of meiosis II (Fig. 4c, d and Supplementary Fig. 5b, c).

The tight coupling of anaphase onset with the loss of DRP1 led us to ask whether DRP1 removal was a prerequisite for, or a consequence of, anaphase onset. To address this question, we performed overexpression and deletion studies to determine the effect of DRP1 on meiosis progression. Microinjection of *Drp1* mRNA 3 h after NEBD delayed polar body extrusion by approximately 1 h and reduced the overall proportion of oocytes that formed a polar body (Fig. 4e). These findings indicate that exogenous DRP1 delays or inhibits polar body extrusion, most likely by delaying the destruction of cyclinB1 and securin^38,39^.

Given these results, depletion of DRP1 is predicted to advance the onset of PB1 extrusion. We first confirmed the optimal timing of DRP1 depletion by Trim-Away and found that DRP1 reaches undetectable levels 4 to 6 h after injection (Supplementary Fig. 6a). In a separate control experiment, similar kinetics of Trim-Away-mediated DRP1 depletion was observed in blastomeres of 2-cell embryos (Supplementary Fig. 6b, c). We used two Trim-Away injection protocols to induce DRP1 degradation: TRIM21/Drp1 or control TRIM21/IgG was injected either 2 h after NEBD (Figs. 4f) or 3 h prior to release from the GV stage (Fig. 4g), allowing for a more extended period of DRP1 clearance. Strikingly, the loss of DRP1 accelerated PB1 extrusion but did not affect the final polar body rate, regardless of injection protocol (Fig. 4f, g). Although PB1 extrusion was slightly faster using the GV injection protocol, the broadly similar time course supports a specific effect of DRP1 depletion on the timing of PB1 extrusion. Taken together, these findings implicate DRP1 in regulating the timing of the MI metaphase-to-anaphase transition.

Cohesion loss upon DRP1 depletion may be a consequence of a defective checkpoint and premature anaphase onset. Alternatively, DRP1 may directly regulate cohesin complex loading maintenance on chromosomes independently of its checkpoint function. To address this question, we depleted DRP1 and examined the kinetochore localisation of SAC proteins MAD1, MAD2, BUB1, and BUBR1 by immunofluorescence. TRIM21/DRP1-mediated depletion of DRP1 caused a dramatic loss of centromeric MAD1 and MAD2 4 h after NEBD, whereas TRIM21/IgG controls showed robust staining for both proteins at the same time point (Fig. 4h–k). In contrast, the localisation of BUB1 and BUBR1 on kinetochores was unaffected by DRP1 depletion (Supplementary Fig. 7a–d). Further, we depleted DRP1 in the presence of the APC/C inhibitor proTAME to prevent premature anaphase onset and examined cohesin localisation. We found that the cohesin subunits REC8 and SMC3 were maintained on chromosome arms in DRP1-depleted oocytes in the presence of proTAME (Supplementary Fig. 7e, f). These data suggest that DRP1 protects cohesion by maintaining SAC activity in meiosis I.

### DRP1 mediates the degradation of cyclinB1 and securin to initiate metaphase-to-anaphase transition

**Fig. 5:**
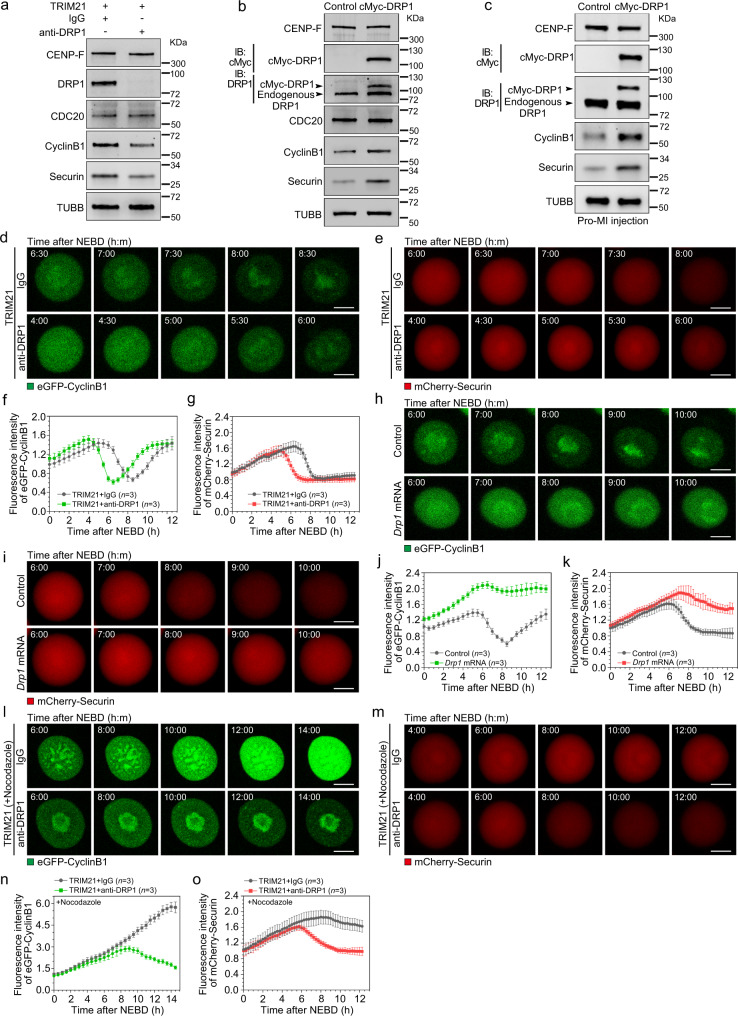
DRP1 loss leads to premature degradation of cyclinB1 and Securin. **a** Premature degradation of cyclinB1 and Securin after DRP1 depletion in oocytes prior to MI stage. Per lysate contains 100 oocytes (NEBD + 6 h). β-Tubulin was used as the loading control. **b**, **c** Accumulation of cyclinB1 and Securin after DRP1 overexpression in GV stage (**b**) or pro-MI stage (**c**) oocytes. Per lysate contains 100 oocytes (NEBD + 7.5 h). β-Tubulin was used as the loading control. **d**, **e** Live-cell imaging of eGFP-cyclinB1 (**d**) and mCherry-Securin (**e**) in control or DRP1 null oocytes. Control and DRP1 null oocytes were injected with *eGFP-cyclinB1* or *mCherry-Securin* mRNA, respectively. See also in Supplementary Movie 1–4. **f**, **g** Time-lapse fluorescence intensities of eGFP-cyclinB1 in (**d**) and mCherry-Securin in (**e**) were measured, respectively. Three independent replicates were performed. **h, i** Live-cell imaging of eGFP-cyclinB1 (**h**) and mCherry-Securin (**i**) in control or DRP1 overexpressed oocytes. Control and DRP1 overexpressed oocytes were injected with *eGFP-cyclinB1* and *mCherry-Securin* mRNA, respectively. See also in Supplementary Movie 5–8. **j**, **k** Time-lapse fluorescence intensities of eGFP-cyclinB1 in (**h**) and mCherry-Securin in (**i**) were measured, respectively. Three independent replicates were performed. **l**, **m** Live-cell imaging of eGFP-cyclinB1 (**l**) and mCherry-Securin (**m**) in control or DRP1 null oocytes cultured with nocodazole. Oocytes were injected with *eGFP-cyclinB1* or *mCherry-Securin* mRNA, and incubated in the medium containing milrinone for 2 h. Then the oocytes were released from milrinone for further maturation in the medium containing nocodazole. See also in Supplementary Movie 9–12. **n**, **o** Time-lapse fluorescence intensities of eGFP-cyclinB1 in (**l**) and mCherry-Securin in (**m**) were measured, respectively. Three independent replicates were performed. Time points after NEBD are indicated as hours: minutes in (**d**, **e**, **h**, **i**, **l** and **m**). Data are presented as the mean ± standard deviation (S.D.). *n* in graphs (**f**, **g**, **j**, **k**, **n** and **o**) refers to the total number of oocytes. Scale bar, 20 μm. NEBD, nuclear envelope breakdown; MI, metaphase I. Representative blots or captures from at least three independent repeats are shown. Source data are provided as a Source Data file. See also in Supplementary Fig. 8.

If DRP1 prevents cohesion loss by maintaining SAC activity, decreasing DRP1 levels would be predicted to accelerate the destruction of cyclinB and securin, triggering anaphase onset^40,41^. Preliminary western blot analyses indicated that the reduction of DRP1 levels in GV stage oocytes using Trim-Away resulted in lower levels of endogenous cyclinB1 and securin at the MI stage (6 h after NEBD) (Fig. 5a). Additionally, we conducted live-cell imaging to monitor the timing of eGFP-cyclinB1 or mCherry-securin in DRP1-depleted oocytes compared to controls. As predicted, TRIM21/DRP1-injected oocytes showed accelerated onset of cyclinB1 and securin destruction (Fig. 5d–g, Supplementary Movie 1–4), consistent with the predicted role of DRP1 in SAC maintenance and the resulting delay of APC/C activity.

Next, we used the same western blot and imaging approaches to test whether overexpression of DRP1 has the inverse effect on cyclinB1 and securin destruction. Indeed, DRP1 overexpression resulted in increased levels of endogenous cyclinB1 and securin at anaphase I onset (7.5 h after NEBD) when these proteins should be close to their nadir (Fig. 5b, c). Imaging experiments confirmed that DRP1 overexpression suppressed cyclinB1 and securin destruction (Fig. 5h–k, Supplementary Movie 5–8). Several control experiments also support the conclusion that DRP1 is acting to maintain the SAC during MI and prevent premature APC/C activation. Namely, the level of the APC/C activator, CDC20, was not changed by DRP1 manipulation (Fig. 5a, b), and overexpression of DRP1 did not alter cyclinB1 or securin levels at 0 or 6 h after NEBD (Supplementary Fig. 8), indicating a specific effect at metaphase-to-anaphase transition.

Next, we probed the relationship between DRP1 and the SAC by asking whether DRP1 depletion can overcome nocodazole-induced SAC activation. In control oocytes, cyclinB1 levels increased continuously after nocodazole treatment, consistent with SAC activation. However, in DRP1-depleted oocytes, cyclinB1 elevation did not continue and instead declined slightly, starting 10 h after NEBD (Fig. 5l, n, Supplementary Movie 9, 10). The securin dynamics were similar to cyclinB1, although securin destruction escaped SAC inhibition more convincingly (Fig. 5m, o, Supplementary Movie 11, 12). The difference in kinetics likely reflects a preference for securin as an APC/C substrate^39^, and therefore securin is more readily destroyed in these experimental conditions. Taken together, these data provide further evidence that DRP1 is essential for maintaining SAC activity, even in the face of nocodazole-induced SAC activation.

### DRP1 regulates APC/C ubiquitin E3 ligase activity by targeting the APC2 subunit

**Fig. 6:**
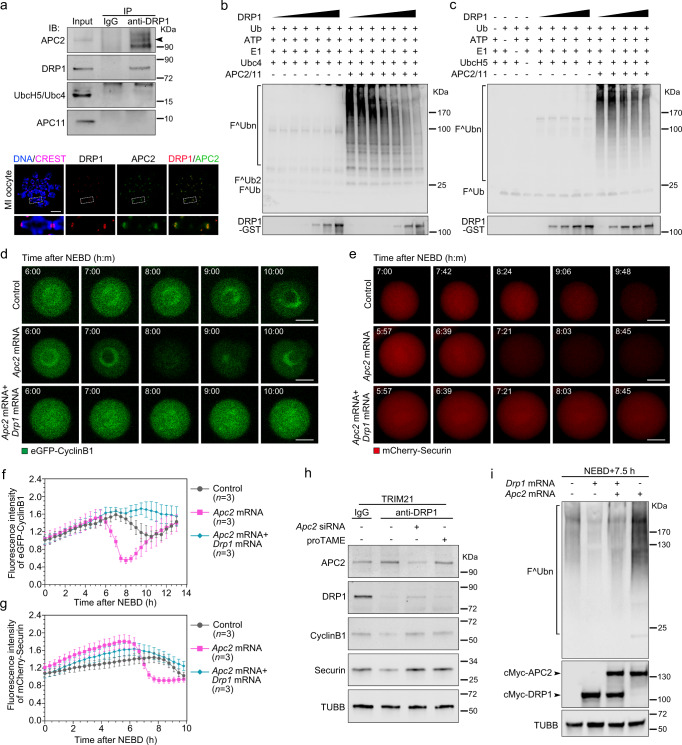
DRP1 inhibits APC/C activity by targeting APC2. **a** Co-IP and immunofluorescence were performed to show the interaction between APC2 and DRP1. Chromosome spreads were stained with DRP1 (red) and APC2 (green). Chromosomes: Hoechst 33342 (blue), kinetochores: CREST (magenta). Enlarged images show representative results in the white dotted frames. Scale bar, 5 μm. **b** Inhibitory effect of the DRP1 on the ubiquitination mediated by APC2/11 and Ubc4 was confirmed with in vitro ubiquitination assay. Purified GST-DRP1 in dilutions of 0.0094, 0.0198, 0.0375, 0.0750, 0.1500, 0.3000 and 0.6000 μg/μl were used in the ubiquitination system. The blots were incubated with anti-FLAG (Ub) and anti-DRP1, respectively. **c** Inhibitory effect of the DRP1 on the ubiquitination mediated by APC2/11 and UbcH5 was confirmed with in vitro ubiquitination assay. Purified GST-DRP1 in dilutions of 0.0375, 0.0750, 0.1500, 0.3000 and 0.6000 μg/μl were used in the ubiquitination system. The blots were incubated with anti-FLAG (Ub) and anti-DRP1, respectively. **d**, **e** Live-cell imaging of eGFP-cyclinB1 and mCherry-Securin in oocytes injected with *Apc2* mRNA or *Apc2* + *Drp1* mRNA. Control, APC2 or APC2 + DRP1 overexpressed oocytes were injected with *eGFP-cyclinB1* (**d**) and *mCherry-Securin* (**e**) mRNA, respectively. Time points after NEBD are indicated as hours: minutes. Scale bar, 20 μm. See also in Supplementary Movie 13–18. **f**, **g** Time-lapse fluorescence intensities of eGFP-cyclinB1 (**d**) and mCherry-Securin (**e**) were measured, respectively. *n* refers to the number of oocytes from three independent replicates. Data are presented as the mean ± standard deviation (S.D.). **h** APC/C defects inhibit premature degradation of cyclinB1 and Securin in DRP1-depleted oocytes. *Apc2* siRNA or proTAME was used to inhibit APC/C activity. Per lysate contains 150 oocytes (NEBD + 6 h). β-Tubulin was used as the loading control. **i** Ubiquitination levels in control, APC2 and APC2 + DRP1 overexpressed oocytes were detected with western blot. The blots were probed with anti-ubiquitin and anti-cMyc, respectively. β-Tubulin was used as the loading control. NEBD, nuclear envelope breakdown. Representative blots, stainings, or captures from at least three independent repeats are shown. Source data are provided as a Source Data file. See also in Supplementary Fig. 9.

Our evidence thus far indicates that DRP1 interfaces with the SAC and contributes to APC/C inhibition. To inhibit APC/C activity, SAC proteins sequester the APC/C activator, CDC20, thereby inhibiting metaphase-to-anaphase transition until the SAC is satisfied and CDC20 is released^11,42–46^. In oocytes, anti-DRP1 co-precipitated APC2 but not APC11 or UbcH5/Ubc4 (Fig. 6a). Moreover, DRP1 co-localised with APC2 on kinetochores (Fig. 6a), providing evidence for an interaction between DRP1 and APC2 in oocytes.

To examine the effect of DRP1 on APC/C activity more directly, we expressed and purified E1, E2, DRP1, APC2, and APC11 with a prokaryotic expression system (Supplementary Fig. 9a–d). We then established in vitro ubiquitination assays using the APC2/11 complex (Supplementary Fig. 9e, f), which forms a two-subunit catalytic core (cullin-RING ligase) that binds E2 to ubiquitinate proteins^47–49^. We found that DRP1 inhibited the E3 ubiquitin ligase activity of APC2/11, and this inhibitory effect was enhanced by increasing the concentration of DRP1 (Fig. 6b, c, and Supplementary Fig. 9g, h). Interestingly, APC2/11 complex ubiquitination was meditated by UbcH5 or Ubc4. However, when UbcH10, UBE2S, Ubc7 or UBE2K was used as the E2 enzyme, no ubiquitination activity was observed in the absence or presence of DRP1 (Supplementary Fig. 9f, i–l).

Loss of APC2 leads to excessive accumulation of cyclinB1^50^ and securin^51^ during meiosis, similar to the effect of DRP1 overexpression. We further examined the ability of APC2 and DRP1 to regulate the APC/C. Upregulating APC2 through injection of *Apc2* mRNA advanced cyclinB1 degradation, an effect that could be prevented by co-injection of *Drp1* mRNA (Fig. 6d, f, Supplementary Movie 13–15). Not surprisingly, securin degradation was subject to the same control mechanisms (Fig. 6e, g, Supplementary Movie 16–18).

Further, we knocked down APC2 in DRP1-depleted oocytes 6 h after NEBD. We found that premature degradation of cyclinB1 and securin were inhibited, which was consistent with the effects of proTAME treatment. These results further suggest that decreased levels of cyclinB1 and securin in DRP1-depleted oocytes are caused by APC2-induced substrate degradation (Fig. 6h). To determine if the inhibition of APC/C activity reflected this DRP1-mediated inhibition of APC2-induced substrate degradation, we performed ubiquitination assays in oocytes 7.5 h after NEBD. We found that APC2 overexpression increased ubiquitination and that this ubiquitination was inhibited by co-overexpression of DRP1 (Fig. 6i). Taken together, our data suggest a model whereby the interaction of DRP1 and APC2 maintains APC/C inhibition, and local destruction of DRP1 relieves APC/C ubiquitination leading to the release of MAD2 and inactivation of the SAC^52,53^.

### Acute changes of DRP1 have no detectable effects on mitochondria during oocyte maturation

Finally, we examined the effect of variation of DRP1 on its canonical role in mitochondrial fission^54^. Transmission electron microscope (TEM) imaging showed that acute loss of DRP1 by the Trim-Away approach did not affect mitochondrial morphology or fission during the oocyte maturation process (Supplementary Fig. 10a). Compared with the controls, overexpression or depletion of DRP1 in GV stage oocytes did not alter mitochondrial distribution or ATP levels (Supplementary Fig. 10b–e) throughout the maturation progress. We evaluated additional cytoplasmic organelles during meiosis maturation and observed no gross differences in the distribution of the Golgi apparatus or endoplasmic reticulum with DRP1 knockdown or overexpression (Supplementary Fig. 10f–i). In addition, during oocytes maturation, neither knockdown nor overexpression of DRP1 affected mitochondrial membrane potential (Supplementary Fig. 11a, b), ROS accumulation (Supplementary Fig. 11c, d), or relative mtDNA copy number (Supplementary Fig. 11e). We also measured the expression of several mitochondrial genes known to be regulated by DRP1 in somatic cells^55^. Our results showed that neither acute DRP1 knockdown nor overexpression affected the expression of *Aldh5a1, Cox10, Mrpl19, Mrpl49, Mterf4, Tmem70*, or *Them4* in MII stage oocytes (Supplementary Fig. 11f–l). Furthermore, we used Mdivi-1, a cell-permeable specific inhibitor to inhibit DRP1 during oocyte maturation. Similar to the results of Trim-Away, there are no detectable changes in mtDNA copy number (Supplementary Fig. 11m). The ratio of phosphorylation of DRP1 at Ser 616 to that at Ser 637, another indicator of the capacity of DRP1-mediated mitochondrial dynamics, was assessed by western blot. The results showed that both DRP1-S616 and DRP1-S637 levels, as well as the DRP1-S616/DRP1-S637 ratio, did not change during oocyte maturation from the GV to MII stage, which may explain the results of knockdown and inhibition (Supplementary Fig. 11n).

## Discussion

**Fig. 7:**
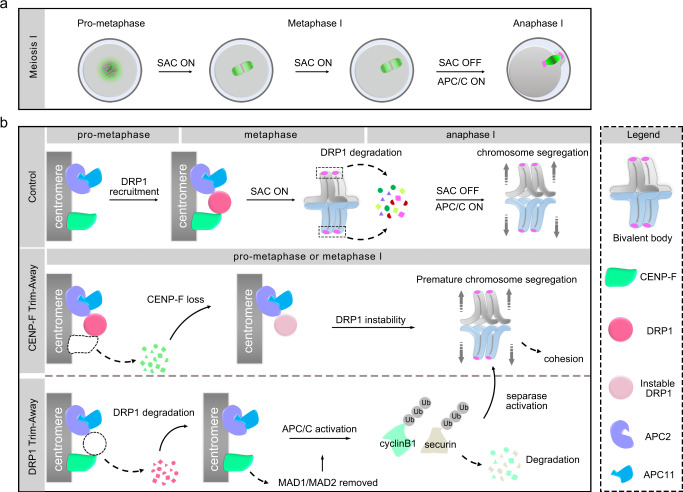
Scheme showing DRP1 regulation of metaphase-to-anaphase transition during oocyte meiosis I. **a** SAC and APC/C regulate mouse oocyte meiotic progression. **b** Centromeric DRP1 is a CENP-F-dependent key regulator in maintaining SAC activity by targeting APC2 to control anaphase entry.

Here, we demonstrate a mechanism responsible for regulating metaphase-to-anaphase transition during oocyte meiosis. Briefly, SAC and APC/C activity control the transition from metaphase to anaphase during meiosis (Fig. 7a). DRP1 is a potential SAC protein recruited to kinetochores by centromeric CENP-F during prometaphase. Centromeric DRP1 binds to APC2 to inhibit its E3 ligase activity, thereby impairing APC/C activity and controlling anaphase entry (Fig. 7b, row 1). CENP-F degradation destabilises centromeric DRP1 (Fig. 7b, row 2). Acute removal of DRP1 prior to anaphase I activates the APC/C by removing MAD1 and MAD2 from kinetochores. Activated APC/C targets and degrades cyclinB1 and securin, which are proteins that inhibit separase. Finally, activated separase cleaves the cohesin links between homologous chromosomes and leads to premature chromosome segregation (Fig. 7b, row 3).

Mitochondrial dynamics are critical regulators of apoptosis, autophagy, and neurodegenerative diseases^56,57^. As a crucial component of the mitochondrial fission machinery, the mitochondrial functions of DRP1 have been well studied in somatic cells. Multiple organelles, including mitochondria, aggregate in DRP1-specific knockout oocytes due to accumulating mitochondrial dysfunction during oocyte growth^23^. A recent study showed that DRP1 knockout leads to elongated and aggregated mitochondria and elevated ROS levels in GV stage oocytes^58^. Given that mitochondrial number increases dramatically from 5 thousand in primary follicles to more than 150 thousand in fully grown oocytes^59^, any conclusions derived from DRP1 knockout models will necessarily involve mitochondria dysfunction. Therefore, to study DRP1 regulation of the transitory metaphase-to-anaphase transition, other approaches, such as Trim-Away, are necessary. To complement the Trim-Away system, Mdivi-1, a selective inhibitor of DRP1 that attenuates mitochondrial division, was used^60^. No detectable variations of mitochondrial copy number were observed when DRP1 was impaired by either Trim-Away or Mdivi-1.

In contrast to growing oocytes, mitochondria in fully grown GV stage oocytes are fragmented, rounded, and almost undivided^61,62^. Mitochondrial fission is typically assessed by two indicators: the DRP1 S616/S637 phosphorylation ratio and mtDNA copy number^63^. Our results showed that these two indicators have no detectable changes during oocyte maturation from the GV to the MII stage. Our results are supported by the fact that mtDNA copy number does not change during oocyte maturation from the GV to MII stage in mice^64,65^, prepubertal sheep^66^, or humans^67,68^. Knockout or overexpression of DRP1 in mouse oocytes does not affect ATP or mtDNA copy numbers^23,58,69^. But these do not exclude the vital role of mitochondria in chromosome segregation since deletions of mtDNA compromise ATP-dependent energy utilisation and resulting chromosomal non-disjunction in human oocytes^70^. Actually, the role of Drp1 in regulating follicle oocyte development via mitochondrial function is not contradictory to its role as a member of SAC in regulating MI-AI transition. The former is attributed to the long-term regulation of DRP1 during follicle oocyte growth; the latter is attributed to the transient effect of DRP1 on oocyte chromosome segregation. Another possibility is that mitochondrial still play essential roles during the MI-AI transition in oocytes. Acute depletion of DRP1 by either Trim-Away or Mdivi-1 could still induce temporary dysfunction of mitochondria that may be responsible for the failure of chromosome separation. Thus, more sensitive approaches are needed for detecting the imperceptible mitochondrial variation during the transient MI-AI transition in oocytes, and to confirm the dual role of DRP1 on both mitochondrial fission and SAC activity.

The function of CENP-F has been debated for a long time. Indeed, the variety of CENP-F functions may be related to its diverse localisation on spindles and centromeres. It has been reported that CENP-F loss activates the SAC and delays the cell cycle^13^. Conversely, another report shows that depletion of CENP-F leads to the loss of the mitotic checkpoint proteins BUBR1 and MAD1 from kinetochores, inhibiting the SAC^19^. These contradictions have been noted previously, but the resolution remains unclear^13^. In our study, CENP-F depletion led to premature loss of cohesion and premature chromosome separation during meiosis. Our results prove that CENP-F is indispensable and maintains SAC activity via its interaction with DRP1. CENP-F ensures that microtubules capture all centromeres and separate chromosomes at the right time.

APC2 (cullin homology protein) and APC11 (RING-finger protein) comprise the minimal ubiquitin ligase module of the APC/C, which catalyses the ubiquitination of cyclinB1 in vitro^48^. Here, we report that DRP1 interacts with the E3 ubiquitin ligase APC2 to regulate the meiotic cell cycle in mouse oocytes. DRP1 inhibited ubiquitination by interacting with APC2, further inhibiting the degradation of key substrates, cyclinB1 and securin of APC/C, thereby preventing metaphase-to-anaphase transition. Our results are consistent with previous studies in which DRP1 overexpression or knockdown induced dynamic changes in cyclinB1^22^, cyclinD1^71^, and cyclinE^22,28^. However, it is still unclear how the loss of DRP1 turns off the SAC. One perspective is that APC/C-mediated ubiquitination drives the disassembly of MAD2 and BUBR1, and then UBCH10 mediates the inactivation of the SAC^52^. In addition, SUMOylation of the APC/C attenuates MCC-mediated suppression of APC/C CDC20 activity, which permits the catalytic activity of the APC/C before the SAC is turned off^72^. As a subunit of APC/C, APC15 catalyses the disassembly/degradation of APC/C-bound MCC^73^. Thus, we conjecture that when all kinetochores are properly attached, DRP1 degradation relieves APC2 inhibition, restoring the catalytic activity of APC2, which is sufficient to remove MAD1 and MAD2 from the APC/C. Coincidentally, DRP1 negatively regulates MARCH5, an OMM-associated E3 ubiquitin ligase that targets MiD49 and MCL1 to control mitochondrial fission and fusion^74^. MARCH5 also binds DRP1 and promotes its ubiquitination^75,76^. This bi-directional relationship implies that if APC2/11 mediates the ubiquitination of DRP1, ubiquitinated DRP1 may negatively regulate APC2 in return. Here, we demonstrate that DRP1 degradation activates anaphase onset, but the detailed mechanism of DRP1 degradation is unclear. Parkin is known to ubiquitinate DRP1 for proteasome-dependent degradation^77^. DRP1 is also the ubiquitinated substrate of APC/C^Cdh1^ ^78^. In addition to ubiquitination, other post-translational modifications, including SUMOylation and phosphorylation, have been reported for DRP1^79^. For example, DRP1 stabilisation is regulated by SUMOylation via the SUMO E3 ligase MUL1^80^, and DRP1 deSUMOylation can be driven by sentrin-specific proteases (SENP3)^81^. Thus, DRP1 degradation during anaphase is likely mediated by post-translational modifications, especially ubiquitination.

Importantly, we define the role of DRP1 in meiosis. In contrast to classical SAC proteins, DRP1 binds APC2 but not CDC20 or CDH1 (the well-known co-activator and receptor of SAC proteins) to regulate APC/C activity. Although some SAC proteins regulate APC/C activity, it is unclear how K-MT attachments drive SAC dissociation from kinetochores. Even when microtubules fail to capture centromeres correctly, the metaphase-to-anaphase transition can proceed and produces aneuploid germ cells. We demonstrate that DRP1 removal from kinetochores can also drive SAC dissociation even when microtubules are not correctly attached to kinetochores. Thus, we suggest that DRP1 degradation is a prerequisite for SAC dissociation from kinetochores before anaphase onset.

In sum, we demonstrate that DRP1 is recruited to kinetochores by centromeric CENP-F during prometaphase and binds to APC2 to inhibit its E3 ligase activity, thereby impairing APC/C activity. Acute removal of DRP1 in oocytes activated APC/C activity and increased cyclinB1 and securin degradation, leading to premature chromosome segregation. Our results illustrate the alternative mechanism of DRP1 in driving APC/C activity during oocyte meiotic maturation.

## Methods

### Mice

C57BL/6 and B6D2 F1 mice were purchased from Inner Mongolia University, China. All mice were maintained in a specific pathogen-free condition in a controlled environment of 20–22 °C and humidity of 40-70%, with a 12/12 h light/dark cycle. All studies adhered to procedures consistent with the National Research Council Guide for the Care and Use of Laboratory Animals and were approved by the Institutional Animal Care and Use Committee at Inner Mongolia University (SYXK 2014-0002 & SYXK 2020-0006).

### Collection of mouse oocytes

Germinal vesicle (GV) stage oocytes were collected from 6-week-old female B6D2 F1 mice by puncturing the follicles of ovaries at 48 h of 5 IU pregnant mare serum gonadotropin (PMSG, SanSheng) injection. In vivo Metaphase II (MII) stage oocytes were collected from the ampullar region 14 h after another 5 IU of human chorionic gonadotropin (hCG, SanSheng) injection. Oocytes were cultured in Chatot-Ziomek-Bavister (CZB) medium covered with mineral oil under the humidified atmosphere of 5% CO_2_ at 37 °C.

### Cell lines

HEK-293 cells (mycoplasma-free) were obtained from American type culture collection (ATCC, CRL-1573) and were cultured in DMEM (GIBCO) supplemented with 10% FBS (ThermoFisher) and 1% penicillin-streptomycin (GIBCO) under the humidified atmosphere of 5% CO_2_ at 37 °C.

### Plasmid construction and acquisition

The mouse *Cenpf, Drp1, Trim21, Apc2* and *Apc11* coding sequences were cloned from cDNA isolated from 6-week-old female C57BL/6 mouse oocytes. For cell transfection or in vitro mRNA transcription, three domains of *Cenpf* with N-terminal *eGFP*, *Drp1* with N-terminal *mCherry* or *cMyc*, *Trim21* with N-terminal *mCherry* or *cMyc*, *Apc2* or *Drp1* with N-terminal *cMyc* were inserted into pCS2+plasmid. The plasmids pBT-*eGFP*-*yclinB1* were gifted from Dr. Zhen-Bo Wang, and pXF6F-*mCherry*-*Securin* was a gift from Dr. Heng-Yu Fan. For protein purification, *Apc2* and *Apc11* coding sequences were cloned into the pET-28a vector, *Drp1* coding sequence was cloned into the pGEX-4T-1 vector. The plasmids pGEX-4T-1-*Ube1*, pET-28a-*Ubch10*, pET-28a-*Ubch5*, pET-28a-*Ubc4*, pET-28a-*Ube2s*, pET-28a-*Ube2k* and pET-28a-*Ubc7* were gifted from Dr. Wei Li.

### ProTAME treatment

GV stage oocytes employed for *Apc2* siRNA or DRP1 Trim-Away were arrested for several hours in the medium containing 2.5 μM milrinone and then released for further maturation in the medium containing 50 μM proTAME (MedChemExpress HY-124955). Samples were collected for immunofluorescence or western blot.

### Transmission electron microscope

Embryos were fixed in 2% glutaraldehyde for 2 h at room temperature, then embedded in 4% low-melting-point agar (Sigma-Aldrich A9045) with the size of 2 × 2 × 2 mm, and stored at 4 °C. The oocytes were then fixed with 1% osmium tetroxide, followed by dehydration in ascending series of ethanol. Ultrathin sections were prepared with a thickness of 70–100 nm. After being stained by lead citrate, the sections were analysed under an electron transmission microscope (FEI Tecnai G2 Spirit Bio TWIN).

### In vitro mRNA synthesis

The linearised plasmids pCS2 + -*cMyc*-*Apc2*, pCS2 + -*cMyc*-*Drp1*, pCS2 + -*cMyc*-*Trim21*, pCS2 + -*mCherry*-*Trim21* and pXF6F-*mCherry*-*Securin* were transcribed in vitro with an mMESSAGE mMACHINE® SP6 Transcription Kit (Thermo Fisher Scientific, AM1340), and pBT-eGFP-*cyclinB1* was transcribed with an mMESSAGE mMACHINE® T3 Transcription Kit (Thermo Fisher Scientific, AM1348). Capped mRNAs were purified with MEGAclear™ Transcription Clean-Up Kit (Thermo Fisher Scientific, AM1908) and then precipitated with ethanol and dissolved in 10 μl of RNase-free water with a stock.

### Co-immunoprecipitation

HEK-293 cells of a 90-mm-dish or 1000 oocytes were lysed in 600 μl IP buffer (20 mM Tris-HCl, 10 mM EDTA, 1 mM EGTA, 150 mM NaCl, 0.05% Triton X-100, 0.05% NP-40, 1 mM PMSF; protease (Sigma-Aldrich P8340, 1:100) and phosphatase inhibitors (Sigma-Aldrich P5726, 1:500) were added before use. Protein A&G agarose beads were washed and then incubated with anti-DRP1, anti-APC2, anti-eGFP or anti-cMyc at 4 °C for 4 h, respectively. Rabbit or mouse IgG was used as the control. Antibody combined beads were collected and incubated with lysates at 4 °C overnight. Beads were washed three times and boiled for 5 min. The SDS page was run for immunoblotting or silver staining, followed by matrix-assisted laser desorption/ionisation time of flight mass spectrometry (MALDI/MS) analysis. The gel samples were sent to the proteomics facility at Nanjing Medical University to undergo MALDI/MS. Original blots can be found in the Source data file.

### Protein purification

*E. coli* Transetta (DE3) cells were transformed with pGEX-4T-1-*Drp1*, pGEX-4T-1-*Ube1*, pET-28a-*Ubch10*, pET-28a-*Ubch5*, pET-28a-*Ubc4*, pET-28a-*Ube2s*, pET-28a-*Ube2k*, pET-28a-*Ubc7*, pET-28a-*Apc2* and pET-28a-*Apc11*, respectively. The protein expression was induced by 0.5 mM IPTG for 16 h at 16 °C. Cell lysates were prepared by repeated freezing-thawing method with liquid nitrogen and water bath of 28 °C. Lysates were incubated with GST Resin (TransGen Biotech DP201-01) or Ni-NTA Resin (TransGen Biotech DP101-01) overnight at 4 °C. GST-DRP1 and GST-UBE1 were eluted with 20 mM GSH. His-UbcH10, His-UbcH5, His-Ubc4, His-UBE2S, His-UBE2K, His-Ubc7, His-APC2 and His-APC11 were eluted with 300 mM imidazole. Eluted proteins were concentrated to 3 mg/ ml with Amicon Ultra-0.5 centrifugal filter units (Millipore). Protein aliquots were frozen in liquid nitrogen immediately and then stored at −80 °C.

### In vitro ubiquitination analysis

The ubiquitin-activating system contains E1 (0.05 μg/μl), E2 (0.1 μg/μl), APC2 (0.5 μg/ μl), APC11 (0.5 μg/μl), FLAG-tagged ubiquitin (0.1 μg/μl), ATP (0.1 M). The reactions were performed in 1×ubiquitination buffer (50 mM Tris-HCl (pH 7.5), 2.5 mM MgCl_2_, 0.05% NP-40, and 0.5 mM dithiothreitol) at 28 °C for 1 h. For the experiment in Fig. 6b, DRP1 concentration was increased from 0.0094 μg/μl to 0.6000 μg/μl. For the experiment in Fig. 6c, DRP1 concentration was increased from 0.0375 μg/μl to 0.6000 μg/μl. The reactions were stopped by adding an equal volume of 2×Laemmli Sample Buffer and boiling for 5 min. The ubiquitylation reactions were resolved by SDS-PAGE on a 4-15% gradient gel, followed by immunoblotting using anti-FLAG. The sixteen-colour image was used to show the bands in visible. Original blots can be found in the Source Data file.

### Trim-Away

Trim-Away is a highly effective method for rapidly and directly degrading the target protein without requiring modification of the genome^33^. The remarkable speed of Trim-Away means that phenotypes can be observed immediately following the degradation of the protein of interest at any stage of a particular biological process. Since the duration for mouse oocyte finishes the transition from prophase to anaphase takes only a few hours, the best way for acute protein degradation is Trim-away, which avoids the compensation effect after long-term gene editing in CKO mice. However, effective of antibodies and off-targets are the obvious limitation for Trim-Away used in oocyte^33^. Thus, an alternative antibody is needed to test the availability and off-targets of Trim-Away.

Purified *Trim21* mRNA was mixed with an indicated antibody, stored at −80 °C and used within one month. The final concentration of *Trim21* mRNA was 1 mg/ml, and the final concentrations of anti-CENP-F (Abcam ab5) and anti-DRP1 (Abcam ab180769) were 1.25 mg/ml and 1.50 mg/ml, respectively. For the GV stage Trim-Away, oocytes were injected with 5 pl *Trim21* mRNA/antibody mix and maintained in the medium containing milrinone for 3 h and then washed out for further development. For pro-MI stage Trim-Away, oocytes matured for 2 h after NEBD were injected with 5 pl *Trim21* mRNA/antibody mix.

### Immunoblotting

Oocytes, HEK-293 cells, Co-IP eluates or ubiquitination reaction products were collected in Laemmli Sample Buffer (Bio-RAD Hercules) containing 1/2000 (v/v) Protease Inhibitor Cocktail and 1/20 (v/v) β-mercaptoethanol (Amresco) and heated at 95 °C for 5 min. Samples were run on 4%-15% Mini-PROTEAN TGX Gels (BIO-RAD) and transferred onto a nitrocellulose membrane. Primary antibodies used were rabbit anti-CENPF (Abcam ab5; 1:500), mouse anti-DRP1 (Abcam ab56788; 1:200), rabbit anti-DRP1 (Ser616) Antibody (Cell Signaling Technology 3455; 1:400), rabbit anti-DRP1 (Ser637) Antibody (Cell Signaling Technology 4867; 1:400), rabbit anti-β-Tubulin (loading Control, Abcam ab6046; 1:500), mouse anti-cMyc (Thermo Fisher R950-25; 1:400), mouse anti-EGFP (Abcam ab184601; 1:400), rabbit anti-cyclinB1 (Cell Signaling 4138; 1:200), goat anti-cyclinB2 (R&D Systems AF6004; 1:400), rabbit anti-FLAG (Sigma-Aldrich F7425; 1:400), rabbit anti-Securin (Cell Signaling 13445; 1:200), rabbit anti-APC2 (Cell Signaling 12301S; 1:200), rabbit anti-APC11 (Abcam ab154546; 1:200), rabbit anti-UbcH5/Ubc4 (Proteintech 28328-AP; 1:200), and rabbit anti-ubiquitin (PTM BIO PTM-1106; 1:200). Secondary antibodies used were Peroxidase-conjugated secondary Goat Anti-Mouse (Jackson Immuno Research Laboratories 115-035-003; 1:2000), Donkey Anti-Rabbit (Jackson 711-035-152; 1:2000), and Donkey Anti-Goat (Jackson 705-035-003; 1:2000). Bands on nitrocellulose membranes were detected using an Enhanced Chemiluminescence Detection Kit (Thermo Fisher Scientific) and captured by the imaging system (Tanon 5200). Where applicable, bands of western blot were quantified with ImageJ (http://rsbweb.nih.gov/ij/). The results of full scan blots were shown in the Source Data file.

### Immunofluorescence

Oocytes were incubated with 0.5% Triton X-100 for 5 min and then fixed with 4% paraformaldehyde for 30 min. Fixed oocytes were blocked in PBS containing 1% BSA, 0.1% Tween-20 and 0.01% Triton X-100 for 1 h. Oocytes were incubated for 48 h at 4 °C with primary antibodies: rabbit anti-CENPF (Abcam ab5; 1:700), mouse anti-Lamin B1 (Abcam ab8982; 1:400), human anti-centromere (Antibodies Incorporated 15-234-0001; 1:500), mouse anti-DRP1 (Abcam ab56788; 1:200), mouse anti-Tubulin (Abcam; 1:1000) rabbit anti-Tubulin (Abcam ab6046; 1:500) and mouse anti-cMyc (Thermo Fisher R950-25; 1:200). Secondary antibodies used were Rhodamine (TRITC) AffiniPure Donkey Anti-Rabbit (Jackson 711-025-152; 1:750), Alexa Fluor 647 AffiniPure Donkey Anti-Human (Jackson 709-605-149; 1:500), and Alexa Fluor 488 AffiniPure Donkey Anti-Mouse (Jackson 715-545-151; 1:500). DNA was stained with 10 μg/ml Hoechst 33342 (Sigma-Aldrich, St. Louis, MO, USA).

### Chromosome spreads

The zona pellucida of oocytes were removed with an acidic M2 medium, and then zona-free oocytes were fixed on the glass slide using chromosome spread solution containing 0.15% Triton X-100, 1% paraformaldehyde and 3 mM dithiothreitol. Immunofluorescent staining was performed after the slides were dried for 12 h. Primary antibodies used were rabbit anti-CENPF (Abcam ab5; 1:50), mouse anti-DRP1 (Abcam ab56788; 1:50), rabbit anti-DRP1 (Abcam ab180769; 1:100), mouse anti-DRP1(BD Biosciences 611112; 1:50), rabbit anti-BUB1 (Abcam ab9000; 1:50), rabbit anti-BUBR1 (Proteintech 115042-AP; 1:100), rabbit anti-APC2 (Proteintech 13559-1-AP; 1:50), rabbit anti-MAD1 (Abcam ab175245; 1:50), rabbit anti-MAD2 (Biolegend 924601; 1:50), human anti-centromere (CREST, Antibodies Incorporated 15-234-0001; 1:100), rabbit anti-REC8 (Proteintech 10793-1-AP; 1:50), rabbit anti-SMC3 (Abcam ab128919; 1:50) and mouse anti-Myc (Thermo Fisher R950-25; 1:100). Secondary antibodies used were Alexa Fluor 647 AffiniPure Donkey Anti-Human (1:50), Rhodamine (TRITC) AffiniPure Donkey Anti-Mouse (Jackson 715-025-150; 1:100), Alexa Fluor 488 AffiniPure Donkey Anti-Mouse (1:100), Alexa Fluor 488 AffiniPure Donkey Anti-Rabbit (Jackson 711-545-152; 1:100), and Rhodamine (TRITC) AffiniPure Donkey Anti-Rabbit (1:100). DNA was stained with 10 μg/ml Hoechst 33342.

### Mitochondria, endoplasmic reticulum (ER) and Golgi apparatus staining

For living oocytes mitochondria, endoplasmic reticulum (ER) and Golgi apparatus staining, different stage oocytes were cultured in CZB medium with mito-tracker (Beyotime C1048; 1:3000), ER-tracker (Beyotime C1041; 1:2000), or Golgi-tracker (Beyotime C1043; 1:2000) at 37 °C for 30 min, respectively, followed by staining with Hoechst 33342 for 5 min. For fixed mitochondria staining, oocytes were stained with the mito-tracker for 20 min before the confocal scanning.

### Drug treatment

Mdivi-1 (Beyotime, SC8028), a selective cell-permeable mitochondrial division Drp1 inhibitor, was used for DRP1 inhibition in mouse oocytes. A concentration of 10 μM Mdivi-1 was used since this is the concentration of IC50 and was proven to have a significant effect on mitochondrial distribution in mouse oocytes^82^. GV stage oocytes were cultured for 4 h in CZB medium supplemented with Mdivi-1 and milrinone, and then cultured for another 12 h in CZB medium containing Mdivi-1 for maturation. DMSO supplemented in CZB medium was used as the negative control. MII stage oocytes were collected for mtDNA copy number measurement.

### Confocal microscopy

Stained oocytes or chromosome spreads were examined with a Nikon A1R microscope using a 100× oil-immersion objective. Optical sections were captured at 2-μm intervals. Images were analysed with NIS-Element AR3.0 software (Nikon). To clearly highlight the location of CENP-F and chromosomes in Supplementary Fig. 1a, the corresponding channels were inverted to black and white by ImageJ software. For live cell imaging, oocytes were cultured in CZB medium under a humidified atmosphere of 5% CO_2_ at 37 °C in the incubator chamber; images were acquired with a Zeiss LSM780 microscope equipped with a ×40 objective. Zeiss Blue software (version 2.3) was used to analyse the data.

### Single oocyte ATP assay

Single oocyte ATP content was determined using an Adenosine 5′-triphosphate (ATP) bioluminescent somatic cell assay kit (Sigma-Aldrich), according to the manufacturer’s instructions. The luminescence intensity was measured using a Luminometer (Promega GloMax® 20/20).

### Measurement of mtDNA copy number

GV stage oocytes were employed for DRP1 Trim-Away or *Drp1* mRNA injection and then matured to MII stage. Oocytes after zona pellucida digesting were incubated in 10 μl lysis buffer (50 mM Tris-HCl, 0.5% Tween-20, 0.1 mM EDTA and 0.15 mg/ml proteinase K) at 55 °C for 1.5 h, followed 95 °C for 10 min. mtDNA copy number was measured by quantifying the unique mitochondrial sequence with the primers: Mito-Forward: 5′-CTA GAA ACC CCG AAA CCA AA-3′; Mito-Reverse: 5′-CCA GCT ATC ACC AAG CTC GT-3′. Beta-2 microglobulin (beta-2M) was used as the internal control (Forward: 5′-ATG GGA AGC CGA ACA TAC TG-3′; Reverse: 5′-CAG TCT CAG TGG GGG TGA AT-3′).

### Measurement of mitochondrial membrane potential

Mitochondrial inner membrane potential dye JC-10 was used to assess mitochondrial membrane potential (ΔΨm). Briefly, oocytes were cultured in the CZB medium with a final concentration of 10 mM JC-10 for 20 min. Then the fluorescence intensities were assessed using confocal microscopy. The mitochondrial membrane potentials were calculated with the ratio of red to green fluorescence intensities.

### ROS Assay

GV stage oocytes were employed for DRP1 trim-away or *Drp1* mRNA injection and then matured to MII stage. MII stage oocytes were incubated in the CZB medium containing 10 mM DCFH-DA for 30 min, and then the fluorescence intensities of oocytes were assessed with confocal microscopy.

### Quantitative real-time PCR

The total oocyte RNA was extracted with the PicoPure™ RNA Isolation Kit (Thermo Fisher). The primers were synthesised by Takara, and the sequences were as follows: *Tmem70* (Forward: 5′-GCG AGC GCA GAT ACC TGT TT-3′; Reverse: 5′-CTG CCA TTT TCT GGC TTA TCA AC-3′); *Mterf4* (Forward: 5′-GGC ACA GCA CAT CAC AGA C-3′; Reverse: 5′-TGT GTG AGT CCC ATC CTG A-3′); *Aldh5a1* (Forward: 5′-CCT GCG TGT AGG TAA CGG ATT T-3′; Reverse: 5′-TGC GTC ATT CAC CTG CTT CT-3′); *Cox10* (Forward: 5′-AGA AGA GCT ATA CAG GGA TTG CC-3′; Reverse: 5′-CTG TGT GAC ATA CAT GCG CTT-3′); *Them4* (Forward: 5′-ACC TAT AAA AAG CCT ATC CCC CT-3′; Reverse: 5′-CCG TTG CCT CTG TGT ATA AAG T-3′); *Mrpl49* (Forward: 5′-GAT AAC CCC GGC TTT GTG GAG-3′; Reverse: 5′-GGC TGC CAA CCA CTA GGA G-3′); *Mrpl19* (Forward: 5′-GGC CCA AGC CGA TTT CAG A-3′; Reverse: 5′-TCA GGA ACC TTC TCT CGT CTT C-3′); *Actin* (Forward: 5′-GTG ACG TTG ACA TCC GTA AAG A-3′; Reverse: 5′-GCC GGA CTC ATC GTA CTC C-3′). RT-PCR was performed with the SYBR Green kit (TaKaRa). The comparative Ct method was used for data analysis, and *Actin* was used as the internal control.

### Quantification of fluorescence intensity

To quantify the fluorescence intensity of REC8 and SMC3 on chromosomes from control and Trim-Away oocytes, imaging was performed on the same microscope using identical imaging parameters. The measuring range on centromeric axes (*C* axes) was confirmed by the two centromeres on one bivalent body, and the measuring range on acentric axes (*A* axes) was confirmed by the connected region of homologous chromosomes. The data were smoothed with Fit splin/LOWESS analysis in GraphPad Prism Software (version 8.0.2). To quantify the fluorescence intensity of cyclinB1 and Securin after Trim-Away treatment, oocytes from control or experimental groups were imaged in the same field to unify the imaging conditions. CyclinB1 or Securin fluorescence densities in the whole oocyte were measured with Zeiss Blue software (version 2.3) and exported to Microsoft Excel (2017).

### Statistics and reproducibility

The data were presented as the mean ± S.D. Statistical analysis was based on the data from at least three biologically independent replicates. Stainings and western blots were repeated at least three times, and representative micrographs, blots or movies from similar results were shown.

Chromosome and kinetochore morphologies, PB1 extrusion, spindle defects, ATP content per oocyte, mtDNA copy number, mitochondrial membrane potential, ROS and mitochondria-related genes expression were analysed using nonparametric Kruskal–Wallis tests in SPSS 19.0 software (IBM). The inter-sister KT distance and fluorescence intensity of MAD1, MAD2, BUB1 and BUBR1 were analysed with two-tailed unpaired Student’s *t* tests for absolute values and were calculated in Microsoft Excel (2017). *P* values are designated as *P < 0.05, **P < 0.01 and ***P < 0.001. The Venn diagram was made following the website http://bioinformatics.psb.ugent.be/webtools/Venn/.

### Reporting summary

Further information on research design is available in the Nature Portfolio Reporting Summary linked to this article.

## Supplementary information

Supplementary Information

Description of Additional Supplementary Files

Supplementary Movie 1. Cyclin B1 degradation in TRIM21+IgG injected oocytes, related to Figure 5d.

Supplementary Movie 2. Cyclin B1 degradation in TRIM21+anti-DRP1 injected oocytes, related to Figure 5d.

Supplementary Movie 3. Securin degradation in TRIM21+IgG injected oocytes, related to Figure 5e.

Supplementary Movie 4. Securin degradation in TRIM21+anti-DRP1 injected oocytes, related to Figure 5e.

Supplementary Movie 5. Cyclin B1 degradation in control oocytes, related to Figure 5h.

Supplementary Movie 6. Cyclin B1 degradation in *Drp1* mRNA injected oocytes, related to Figure 5h.

Supplementary Movie 7. Securin degradation in control oocytes. Related to Figure 5i.

Supplementary Movie 8. Securin degradation in *Drp1* mRNA injected oocytes. Related to Figure 5i.

Supplementary Movie 9. Cyclin B1 degradation of TRIM21+IgG injected oocytes cultured in nocodazole, related to Figure 5l.

Supplementary Movie 10. Cyclin B1 degradation of TRIM21+anti-DRP1 injected oocytes cultured in nocodazole, related to Figure 5l.

Supplementary Movie 11. Securin degradation of TRIM21+IgG injected oocytes cultured in nocodazole, related to Figure 5m.

Supplementary Movie 12. Securin degradation of TRIM21+anti-DRP1 injected oocytes cultured in nocodazole, related to Figure 5m.

Supplementary Movie 13. Cyclin B1 degradation in control oocytes, related to Figure 6d.

Supplementary Movie 14. Cyclin B1 degradation in *Apc2* mRNA injected oocytes, related to Figure 6d.

Supplementary Movie 15. Cyclin B1 degradation in *Apc2*+*Drp1* mRNA injected oocytes, related to Figure 6d.

Supplementary Movie 16. Securin degradation in control oocytes, related to Figure 6e.

Supplementary Movie 17. Securin degradation in *Apc2* mRNA injected oocytes, related to Figure 6e.

Supplementary Movie 18. Securin degradation in *Apc2*+*Drp1* mRNA injected oocytes, related to Figure 6e.

Reporting Summary

## Data Availability

Source data are provided with this paper. The mass spectrometry proteomics data related to Supplementary Fig. 2b–d generated in this study have been deposited into the Zenodo database under accession code 4603598. The DOI is 10.5281/zenodo.4603598^83^. All data are available from the corresponding author upon reasonable request. Source data are provided with this paper.

## Source data

Source Data
