# Supplementary material for: CENP-F-dependent DRP1 function regulates APC/C activity during oocyte meiosis I
Source: Nat Commun. 2022 Dec 13;13:7732. doi: 10.1038/s41467-022-35461-5 (PMC9747930; doi:10.1038/s41467-022-35461-5)
Supplement: Supplementary file 1 — Supplementary Information [file 41467_2022_35461_MOESM1_ESM.pdf]

Supplementary Information for

CENP-F-dependent DRP1 function regulates APC/C activity during oocyte meiosis I

Cheng-Jie Zhou[1*], Xing-Yue Wang[1], Yan-Hua Dong[1], Dong-Hui Wang[1], Zhe Han[1], Xiao-Jie Zhang[1], Qing-Yuan Sun[2], John Carroll[3], and Cheng-Guang Liang[1*]

This PDF file includes the following:

Supplementary Figure 1-10 and Supplementary Figure Legends

Supplementary Figures

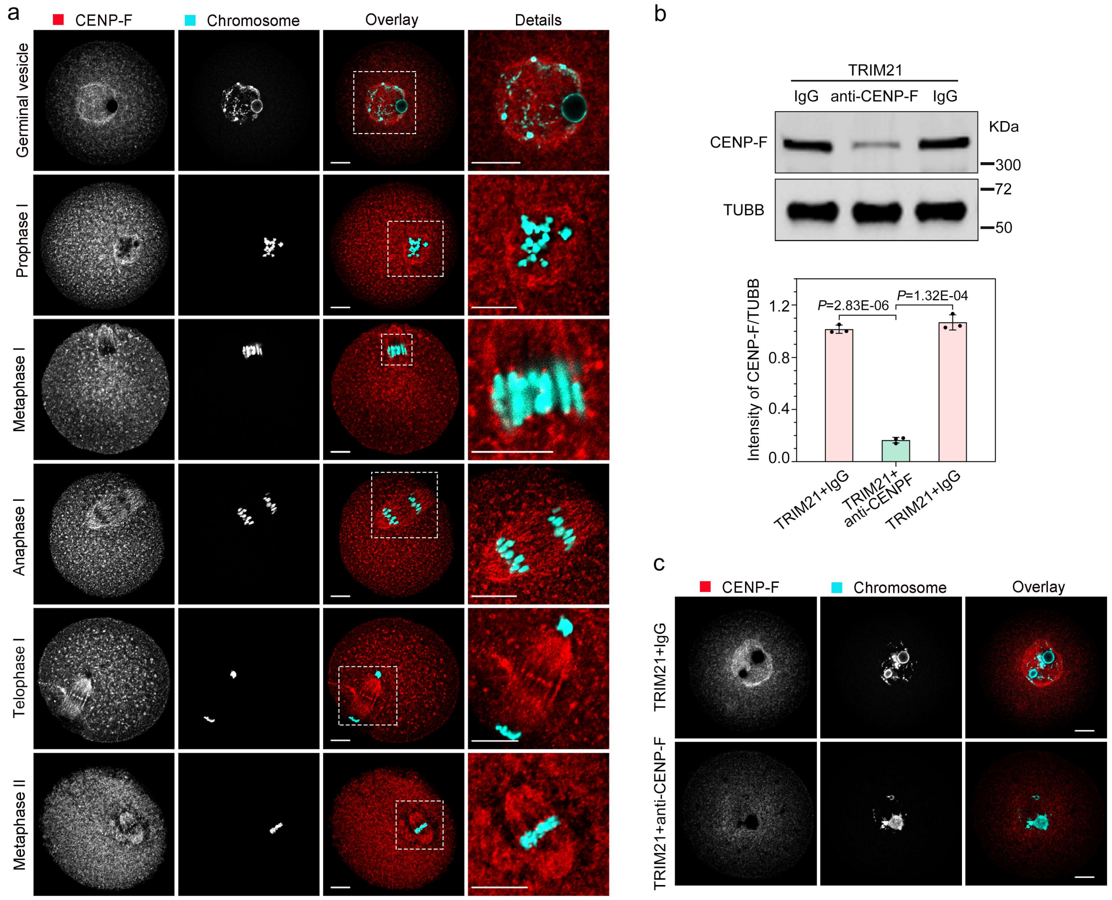

Supplementary Figure 1. CENP-F localisation and Trim-Away effect during mouse oocyte meiotic maturation, related to Figure 1.

a Oocytes at the Germinal vesicle (GV), Prophase I, Metaphase I, Anaphase I, Telophase I, and Metaphase II were stained with anti-CENP-F (white in column 1 and red in columns 3 and 4). Chromosomes were stained with Hoechst 33342 (white in column 2 and turquoise in columns 3 and 4). The white dotted frames indicate the region shown in detail. Scale bar, 15 µm.

b, c CENP-F depletion with the Trim-Away approach was confirmed with western blot (b) and immunofluorescence (c). GV stage oocytes injected with TRIM21+IgG or TRIM21+anti-CENP-F were maintained in the medium containing milrinone for 2 h and then collected for blotting or staining. The blots were incubated with anti-CENP-F. β-Tubulin was used as the loading control. Data are presented as the mean ± standard deviation (S.D.). *P* values were calculated based on nonparametric Kruskal-Wallis tests. GV stage oocytes were stained with anti-CENP-F (white in column 1 and red in column 3). Chromosomes were stained with Hoechst 33342 (white in column 2 and turquoise in column 3).

Scale bar, 15 μm. Representative stainings or blots from at least three independent repeats are shown. The blots from three independent repeats were quantified with ImageJ. Source data are provided as a Source Data file.

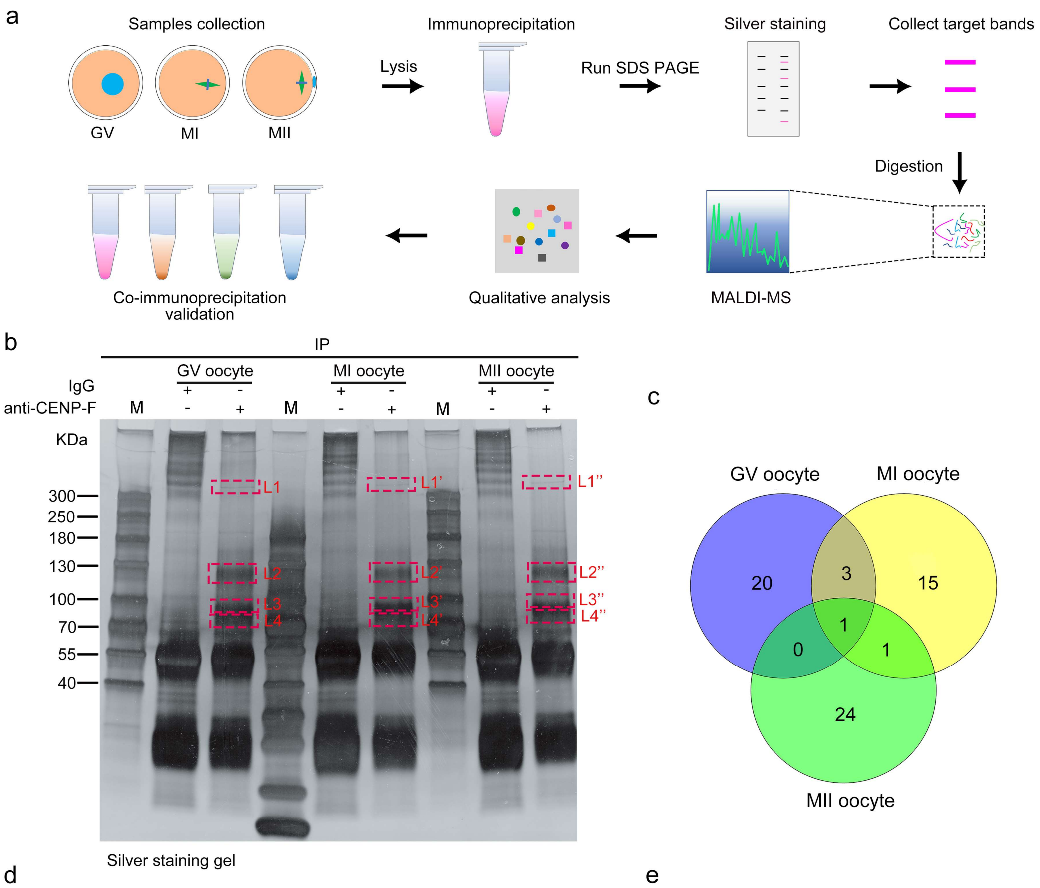

d

Protein Name	Accession No.	Score	Peptides numbers
DRP1	BAC06576	252	25
PCDH9	NP_001258728	210	25
Jumonji	NP_068678	181	31
SPEG	Q62407	175	27
MAP4	NP_001298093	175	22
AP3D1	EDL31507	173	22
PTK7	EDL23512	128	19
Dynein light chain 1	NP_083097	48	7

e

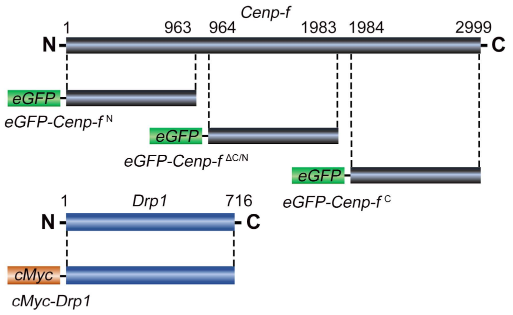

Supplementary Figure 2. Identification of CENP-F interacting proteins with MALDI/MS, related to Figure 2.

a Scheme of the experimental procedure to identify CENP-F interacting proteins. Samples of GV, MI or MII stage oocytes were collected and lysis for immunoprecipitation. Silver staining was performed after SDS-PAGE. The target bands were collected and digested for matrix-assisted laser desorption/ionisation time of flight mass spectrometry (MALDI/MS). Results of MALDI/MS were subjected to qualitative analysis, and co-immunoprecipitation was performed for protein interaction validation.

b Silver staining of SDS-PAGE after CENP-F IP. The red dotted frames indicate the differential bands compared with the IgG group, which were subjected to collection and digestion. Representative blot from three independent repeats with similar results is shown.

c The Venn diagram of identified proteins in GV, MI and MII stage oocytes.

d The list of CENP-F interacting proteins identified in oocytes by MALDI/MS.

e Mapping of the DRP1 interaction domain on CENP-F. *Cenp-f* fragments were fused with *eGFP*; *Drp1* was fused with *cMyc*. Both sequences of *Cenp-f* fragments and *Drp1* were cloned into the pCS2+ plasmid. GV, germinal vesicle; MI, metaphase I; MII, metaphase II. Source data are provided as a Source Data file.

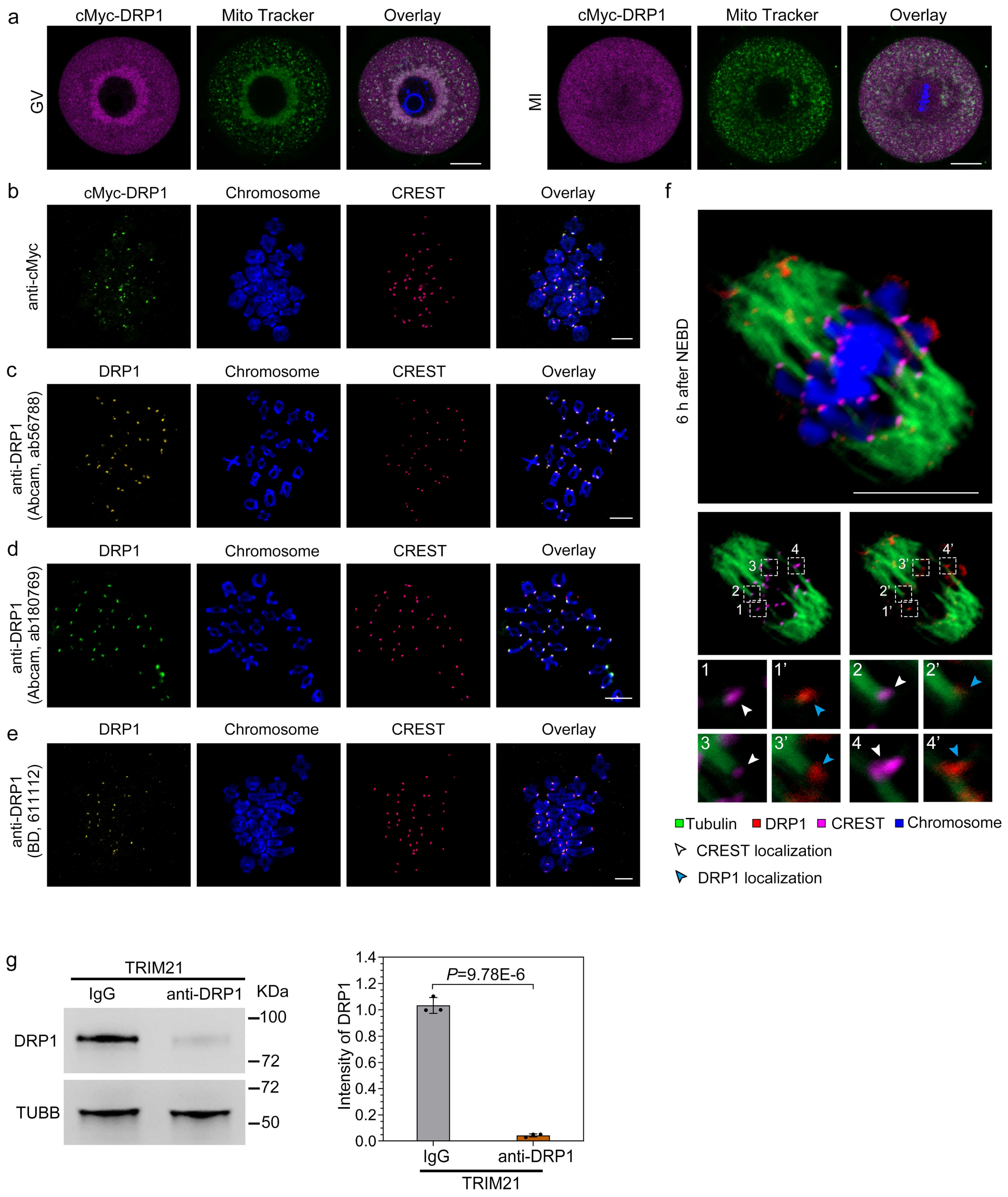

Supplementary Figure 3. Identification of DRP1 localisation on the kinetochores, related to Figure 2.

a Co-localisation of cMyc-DRP1 and mitochondria in GV and MI stage oocytes. GV stage oocytes were injected with *cMyc-Drp1* mRNA, maintained in the medium containing milrinone for 4 h, and then released for further maturation. GV and MI stage oocytes were collected for staining with anti-cMyc (purple) and mitochondria tracker (green). Scale bar, 20 μm.

b Localisation of exogenous DRP1 fused cMyc (green). Chromosome spreads were strained with the cMyc antibody. Scale bar, 5 μm.

c-e DRP1 localisation (yellow in c and e, green in d) on kinetochores was detected with anti-DRP1 antibodies purchased from different vendors. Scale bar, 5 μm.

f DRP1 co-localised with CREST at the end of the microtubule. Oocytes at the MI stage were fixed and stained with DRP1 (red), Tubulin (green), and CREST (magenta). White arrows (1, 2, 3 and 4) indicate the kinetochores. Azure arrows (1', 2', 3' and 4') indicated the DRP1. The white dotted frames indicate the region shown in detail. Scale bar, 5 μm.

g GV stage oocytes injected with TRIM21+IgG or TRIM21+anti-DRP1 were maintained in the medium containing milrinone for 4 h. Oocyte lysates were immunoblotted for DRP1. β-Tubulin was used as the loading control. The blots from three independent repeats were quantified with ImageJ. Data are presented as the mean ± standard deviation (S.D.). *P* values were calculated based on unpaired Student's t-test (two-tailed).

Chromosomes were stained with Hoechst 33342 (blue), and kinetochores were marked with CREST (red in b, c, d, e and magenta in f). GV, germinal vesicle; NEBD, nuclear envelope breakdown; MI, metaphase I. Representative stainings or blots from at least three independent repeats are shown. Source data are provided as a Source Data file.

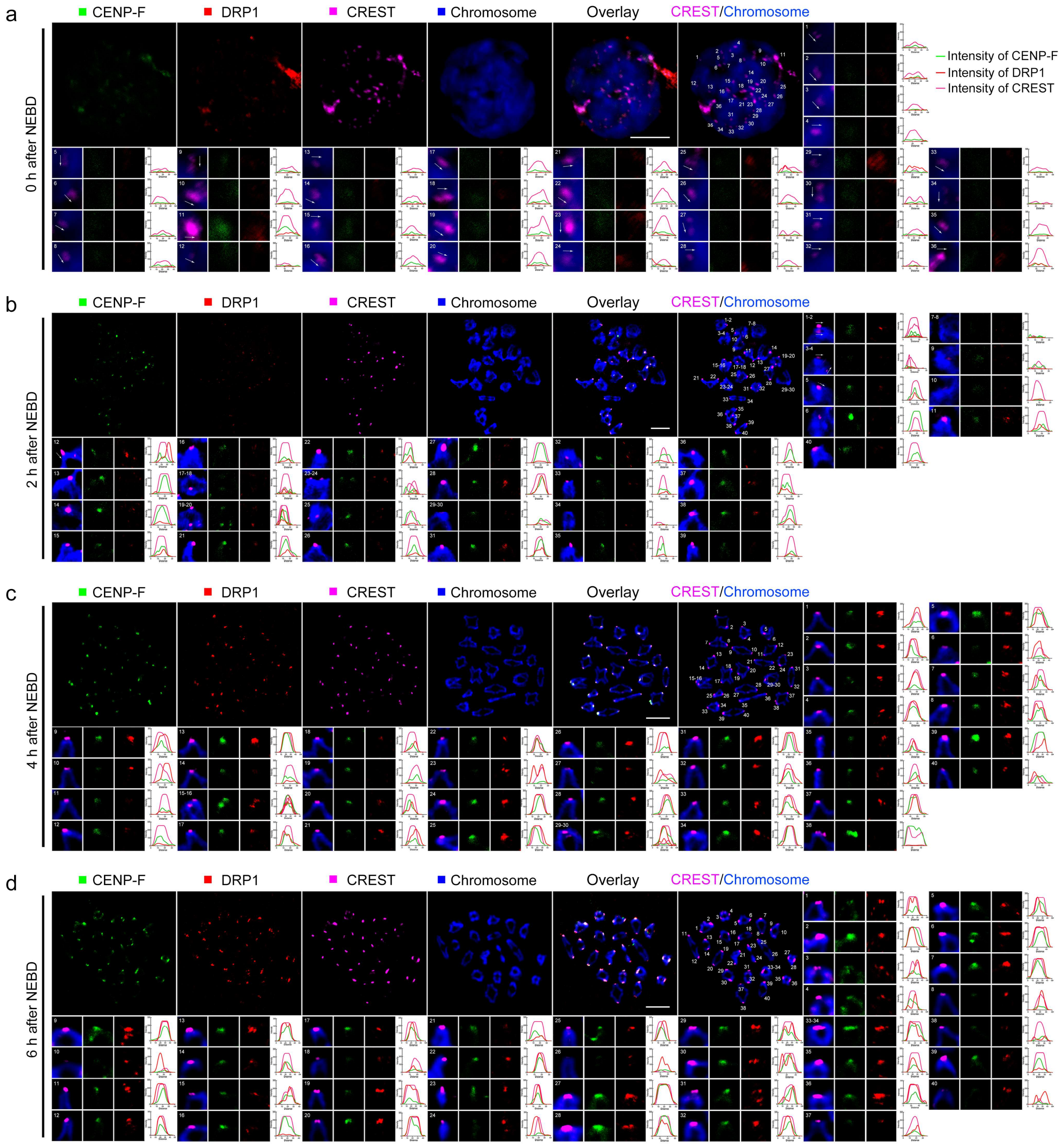

Supplementary Figure 4. Dynamics of DRP1 and CENP-F on the kinetochores during prophase I to metaphase I transition, related to Figure 2.

a-d Localisation of CENP-F and DRP1 on kinetochores at 0 h (a), 2 h (b), 4 h (c), and 6h (d) after NEBD. Chromosome spreads were stained with CENP-F (green), DRP1 (red), and CREST (magenta). Chromosomes were stained with Hoechst 33342 (blue). White arrows indicate the measurement direction of the fluorescence intensity. If not defined, the measurement direction starts from the left of the kinetochore to the right in the inserts. Fluorescence intensities of CENP-F (green line), DRP1 (red line) and CREST (magenta line) are shown in the line graph. The distance showed in pixel. Scale bar,

5 μm. NEBD, nuclear envelope breakdown. Representative stainings from at least three independent repeats are shown. Source data are provided as a Source Data file.

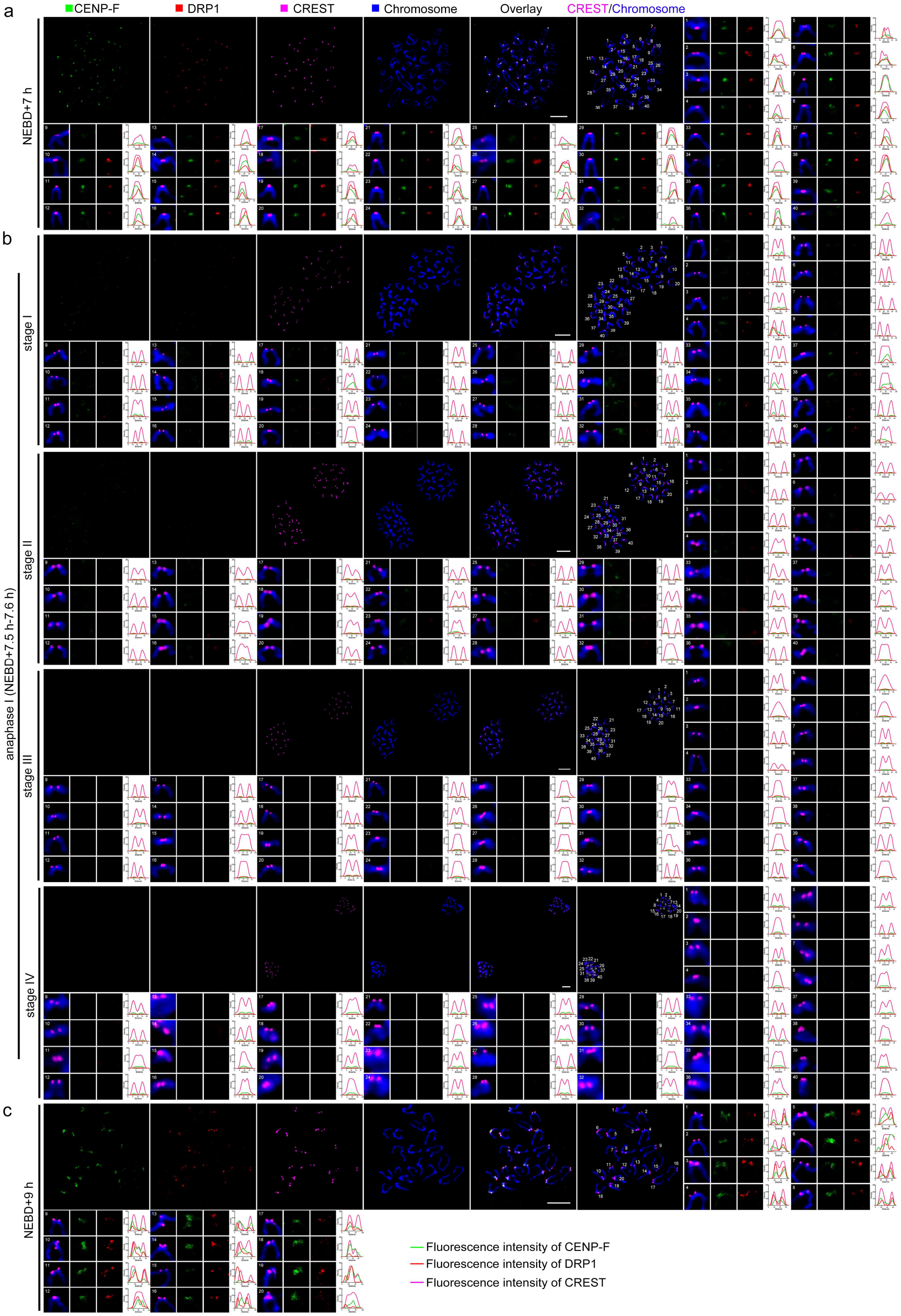

a
CENP-F
DRP1
CREST
Chromosome
Overlay
CREST/Chromosome
NEBD+7 h
b
anaphase I (NEBD+7.5 h-7.6 h)
stage I
stage II
stage III
stage IV
c
NEBD+9 h
Fluorescence intensity of CENP-F
Fluorescence intensity of DRP1
Fluorescence intensity of CREST

Supplementary Figure 5. Dynamics of DRP1 and CENP-F on the kinetochores during metaphase I to anaphase I transition, related to Figure 4.

a-c Localisation of CENP-F and DRP1 on kinetochores at 7 h (a), 7.5-7.6 h (b) and 9 h (c) after NEBD. Stages I-IV of anaphase were determined by the distance of homologous chromosomes after chromosome spreads. Chromosome spreads were stained with CENP-F (green), DRP1 (red) and CREST (magenta). Chromosomes were stained with Hoechst 33342 (blue). White arrows indicate the measurement direction of the fluorescence intensity. If not defined, the measurement direction starts from the left of the kinetochore to the right in the inserts. Fluorescence intensities of CENP-F (green line), DRP1 (red line) and CREST (magenta line) are shown in the line graph. The distance showed in pixel. Scale bar, 5 μm. NEBD, nuclear envelope breakdown. Representative stainings from at least three independent repeats are shown. Source data are provided as a Source Data file.

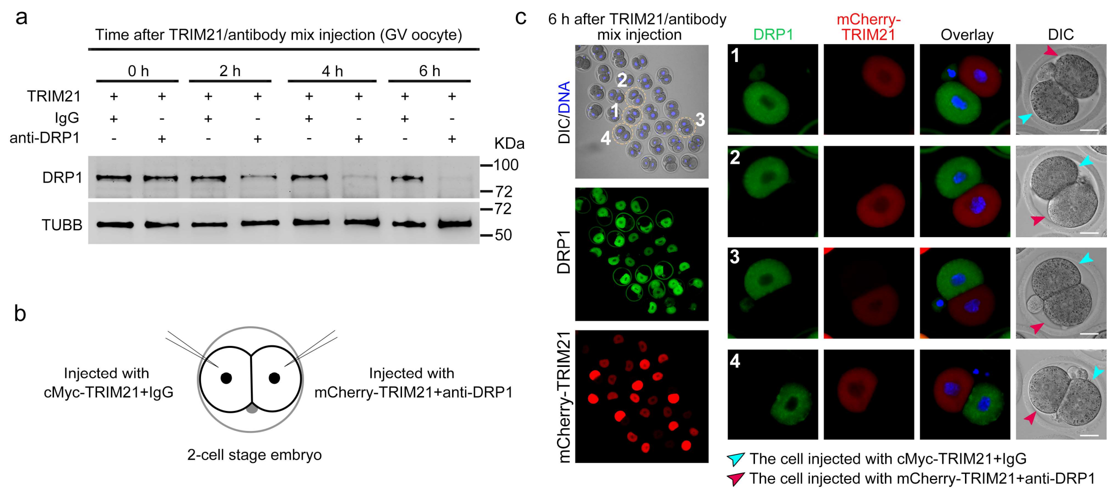

Supplementary Figure 6. The efficiency of Trim-Away approach in degrading DRP1, related to Figure 4.

a The GV stage oocytes injected with TRIM21+IgG or TRIM21+anti-DRP1 were maintained in the medium containing milrinone for 0 h, 2 h, 4 h and 6 h, respectively. Oocyte lysates were immunoblotted for DRP1. β-Tubulin was used as the loading control.

b Schematic of Trim-Away approach in 2-cell stage embryo. cMyc-TRIM21+IgG was injected into one blastomere as the negative control. Meanwhile, mCherry-TRIM21+anti-DRP1 was injected into another blastomere.

c Images of 2-cell stage embryos injected with cMyc-TRIM21+IgG and mCherry-TRIM21+anti-DRP1 followed the protocol shown in (b). Embryos were stained with anti-DRP1 (green). mCherry-TRIM21 is shown in red. Dashed circled embryos with the labelled number (1, 2, 3, and 4) were amplified as the representative. DNA was stained with Hoechst 33342 (blue). Scale bar, 20 μm. GV, germinal vesicle. Representative blots or stainings from at least three independent repeats are shown. Source data are provided as a Source Data file.

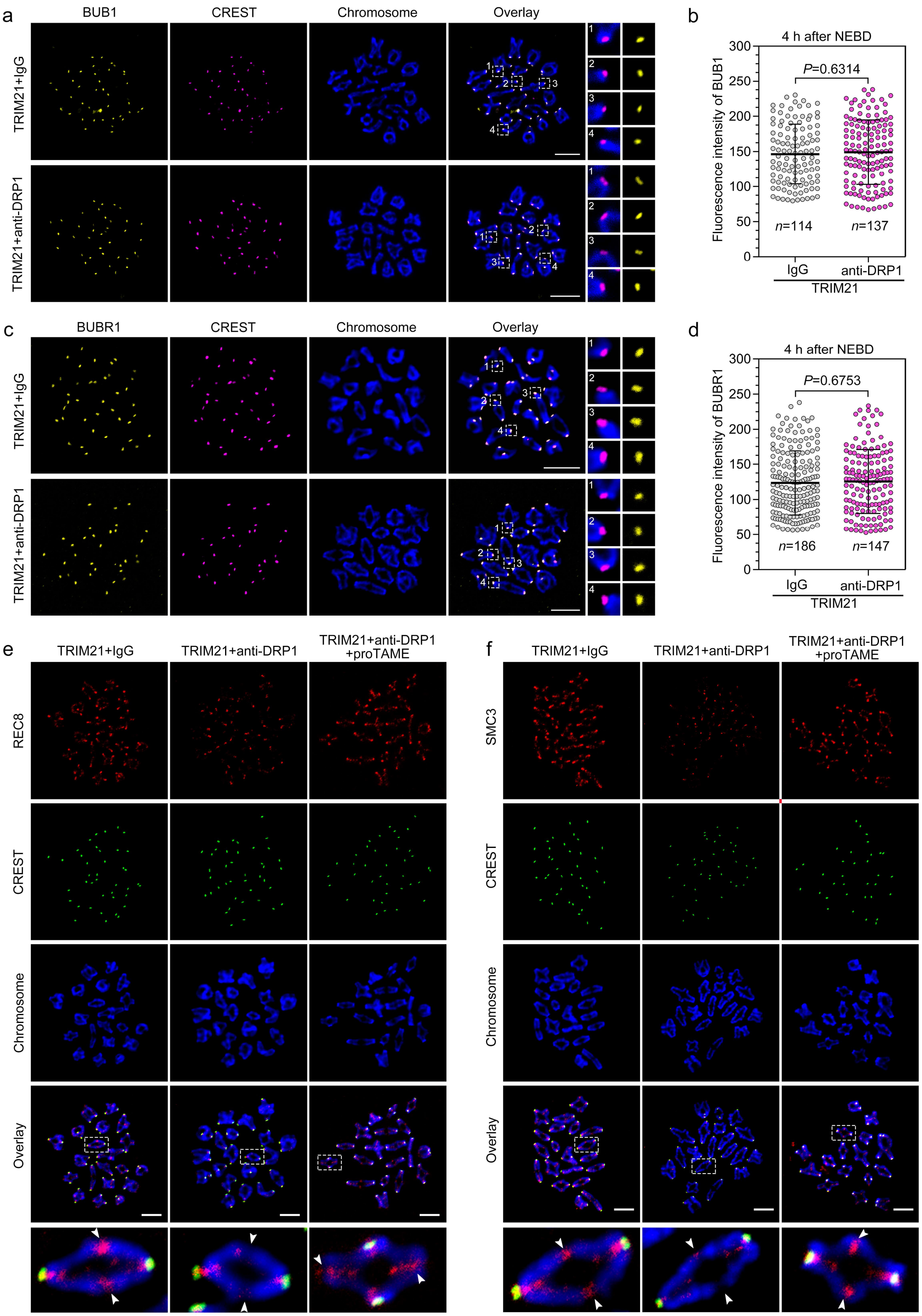
a
BUB1
CREST
Chromosome
Overlay
TRIM21+IgG
TRIM21+anti-DRP1
b
4 h after NEBD
P=0.6314
Fluorescence intensity of BUB1
n=114
n=137
IgG
anti-DRP1
TRIM21
c
BUBR1
CREST
Chromosome
Overlay
TRIM21+IgG
TRIM21+anti-DRP1
d
4 h after NEBD
P=0.6753
Fluorescence intensity of BUBR1
n=186
n=147
IgG
anti-DRP1
TRIM21
e
TRIM21+IgG
TRIM21+anti-DRP1
TRIM21+anti-DRP1 +proTAME
REC8
CREST
Chromosome
Overlay
f
TRIM21+IgG
TRIM21+anti-DRP1
TRIM21+anti-DRP1 +proTAME
SMC3
CREST
Chromosome
Overlay

Supplementary Figure 7. DRP1 loss does not affect BUB1 and BUBR1, related to Figure 4.

a The GV stage oocytes injected with TRIM21+IgG or TRIM21+anti-DRP1 were maintained in the medium containing milrinone for 3 h and then released for further maturation. Oocytes collected 4 h after NEBD were stained for BUB1 (yellow). Enlarged images show representative results.

b Fluorescence intensity of BUB1 in (a) was measured.

c The GV stage oocytes injected with TRIM21+IgG or TRIM21+anti-DRP1 were maintained in the medium containing milrinone for 3 h and then released for further maturation. Oocytes collected 4 h after NEBD were stained for BUBR1 (yellow). Enlarged images show representative results.

d Fluorescence intensity of BUBR1 in (c) was measured.

e, f DRP1-depleted oocytes were cultured for 6 h in the medium with proTAME. Oocytes were stained for REC8 (red in e) and SMC3 (red in f). White arrows indicate the localisation of REC8 and SMC3 on the acentric axes.

Chromosomes were stained with Hoechst 33342 (blue), and kinetochores were marked with CREST (magenta in a and c, green in e and f). Data are presented as the mean ± standard deviation (S.D.). *P* values were calculated based on unpaired Student's t-test (two-tailed). *n* in graphs refers to the total number of kinetochores in three independent replicates. The white dotted frames indicate the region shown in detail. Scale bar, 5 μm. GV, germinal vesicle; NEBD, nuclear envelope breakdown. Representative staining from at least three independent repeats is shown. Source data are provided as a Source Data file.

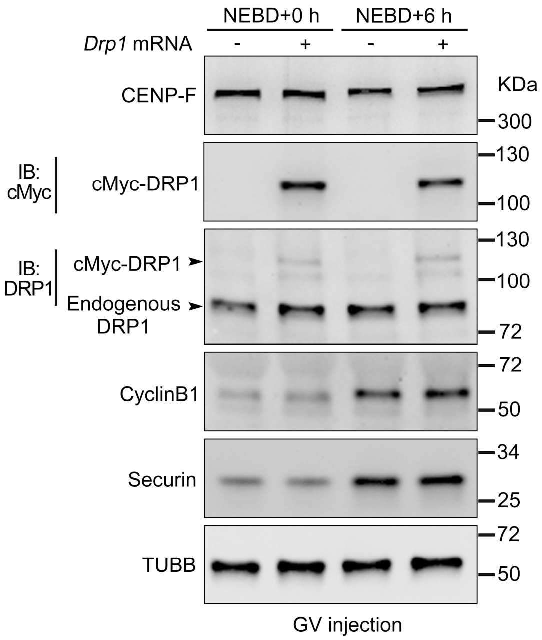

Supplementary Figure 8. CyclinB1 and Securin levels after DRP1 overexpression, related to Figure 5.

Western blot showing protein levels of cyclinB1 and Securin after DRP1 overexpression at the GV stage. Oocytes collected at 0 h and 6 h after NEBD were used for western blot. Per lysate containing 100 oocytes was incubated with anti-CENP-F, anti-DRP1, anti-cMyc, anti-cyclinB1 and anti-Securin, respectively. β-Tubulin was used as the loading control. GV, germinal vesicle; NEBD, nuclear envelope breakdown. Representative blots from three independent repeats are shown. Source data are provided as a Source Data file.

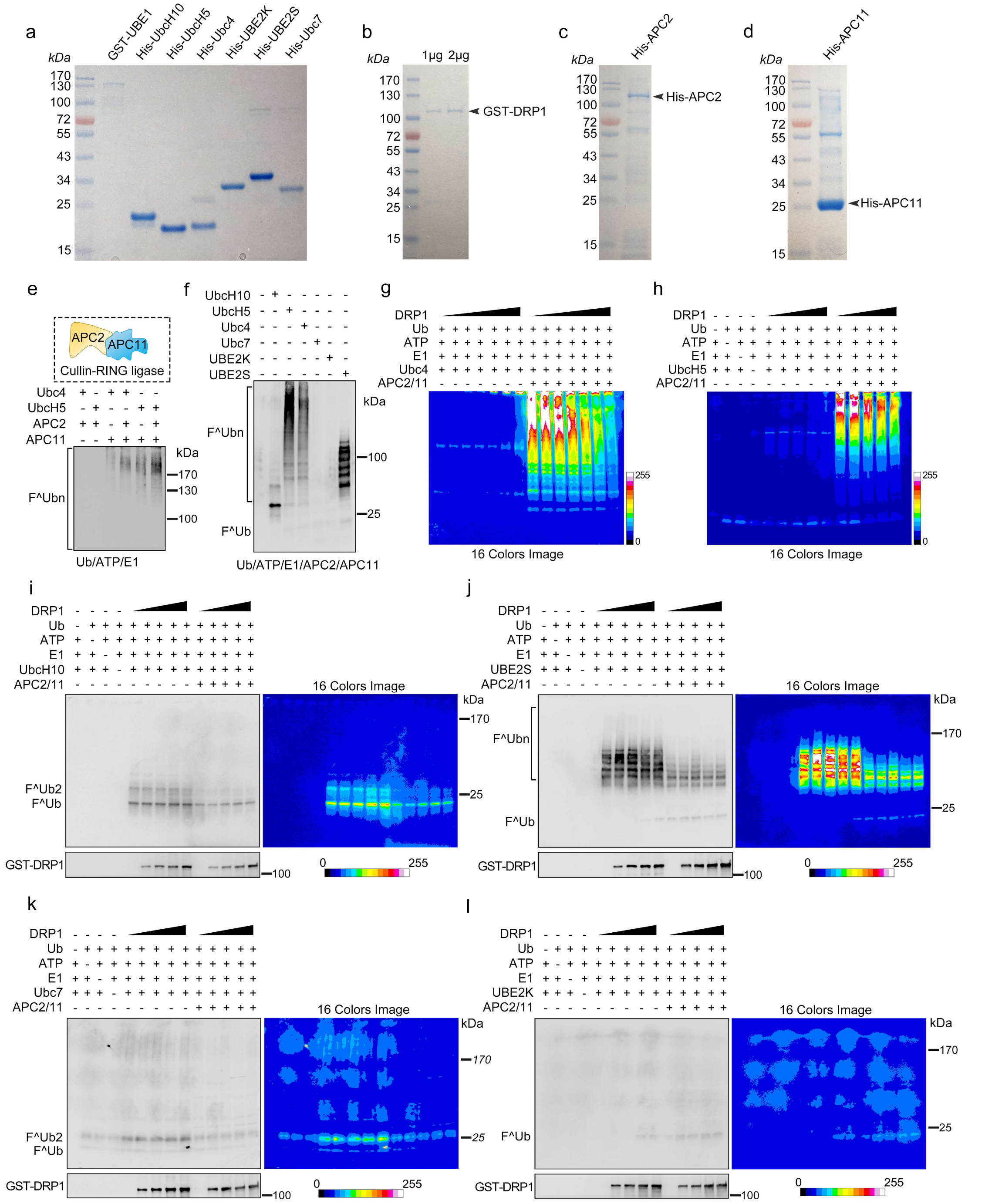

Supplementary Figure 9. Inhibitory effect of the DRP1 on the ubiquitination mediated by different E2 enzymes, related to Figure 6.

a Recombinant E1 (GST-BUE1) and E2 enzymes (His-UbcH10, His-UbcH5, His-Ubc4, His-UBE2K, His-UBE2S, and His-Ubc7) were purified and used for SDS-PAGE, followed by the Coomassie staining.

b-d Coomassie staining of purified GST-DRP1, His-APC2 and His-APC11.

e APC2 and APC11 form a two-subunit catalytic core (Cullin-RING ligase). The activity of Cullin-

RING ligase was meditated by Ubc4 and UbcH5. APC2 enhanced the activity of APC11. The blots were incubated with anti-FLAG (Ub).

f E2 enzymes meditated APC2/11 ubiquitination. The blots were incubated with anti-FLAG (Ub).

g, h The sixteen-colour image of Figure 6b and c.

i-l Inhibitory effects of DRP1 on the ubiquitination mediated by APC2/11 and UbcH10, UBE2S, Ubc7 and UBE2K, respectively, were confirmed with *in vitro* ubiquitination assay. Purified GST-DRP1 in dilutions of 0.0375, 0.0750, 0.1500, 0.3000 and 0.6000 μg/μl, respectively, were contained in the ubiquitination system. Per reaction containing 10 μl was loaded for western blot. The blots were incubated with anti-FLAG (Ub) and anti-DRP1, respectively. Representative blots from at least three independent repeats are shown. Source data are provided as a Source Data file.

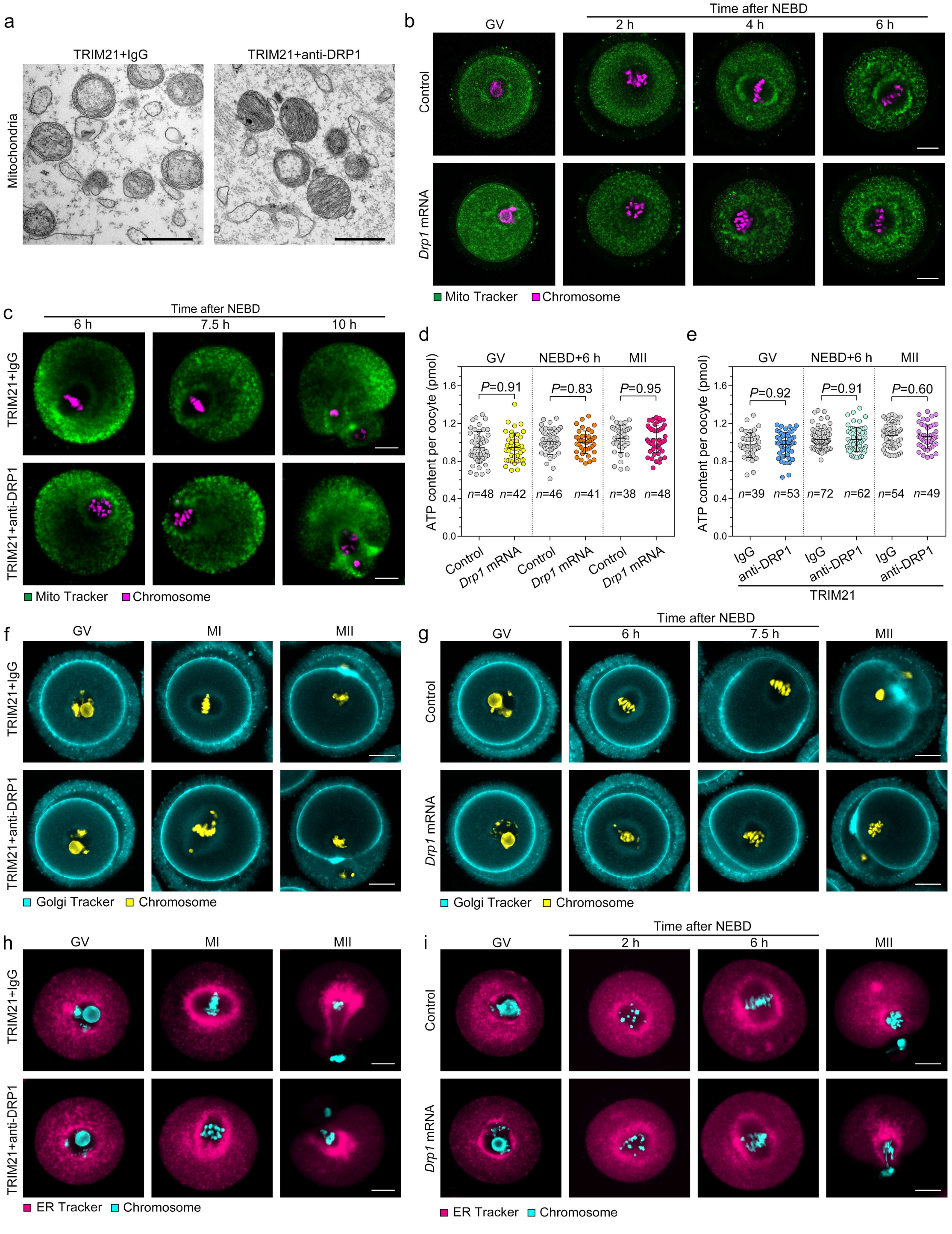

Supplementary Figure 10. Acute variation of DRP1 expression has no detectable effects on mitochondrial dynamics during GV to MII transition in oocytes.

a Mitochondria morphotype in control and DRP1-depleted MII stage oocytes. The GV stage oocytes injected with TRIM21+IgG or TRIM21+anti-DRP1 were maintained in the medium containing milrinone for 3 h, then matured to the MII stage for transmission electron microscope observation. Scale

bar, 500 nm.

b Mitochondria distribution in control or DRP1 overexpressed oocytes. Oocytes at different stages were stained with Mito-Tracker (green).

c Mitochondria distribution in TRIM21+IgG or TRIM21+anti-DRP1 injected oocytes. Oocytes at different stages were stained with Mito-Tracker (green).

d The GV stage oocytes injected with *Drp*1 mRNA were maintained in the medium containing milrinone for 3 h and then released for further maturation. ATP content per oocyte was measured.

e The GV stage oocytes injected with TRIM21+IgG or TRIM21+anti-DRP1 were maintained in the medium containing milrinone for 3 h and then released for further maturation. ATP content per oocyte was measured.

f The Golgi apparatus dynamics in TRIM21+IgG or TRIM21+anti-DRP1 injected oocytes. Oocytes from different maturation stages were stained with Golgi-Tracker (turquoise).

g The Golgi apparatus dynamics in control or DRP1 overexpressed oocytes. Oocytes from indicated culture times or stages were stained with Golgi-Tracker (turquoise).

h ER dynamics in TRIM21+IgG or TRIM21+anti-DRP1 injected oocytes. Oocytes from different maturation stages were stained with ER-Tracker (crimson).

i ER dynamics in control or DRP1 overexpressed oocytes. Oocytes from indicated culture times or stages were stained with ER-Tracker (crimson).

Chromosomes were stained with Hoechst 33342 and exhibited in pink (b, c), yellow (f, g) or blue (h, i). Scale bar in b, c and f-i, 20 μm. *n* in graphs refers to the total number of oocytes in three independent replicates. Data are presented as the mean ± standard deviation (S.D.). *P* values were calculated based on unpaired Student's t-test (two-tailed). GV, germinal vesicle; NEBD, nuclear envelope breakdown; ER, endoplasmic reticulum. Representative micrographs from at least three independent repeats are shown. Source data are provided as a Source Data file.

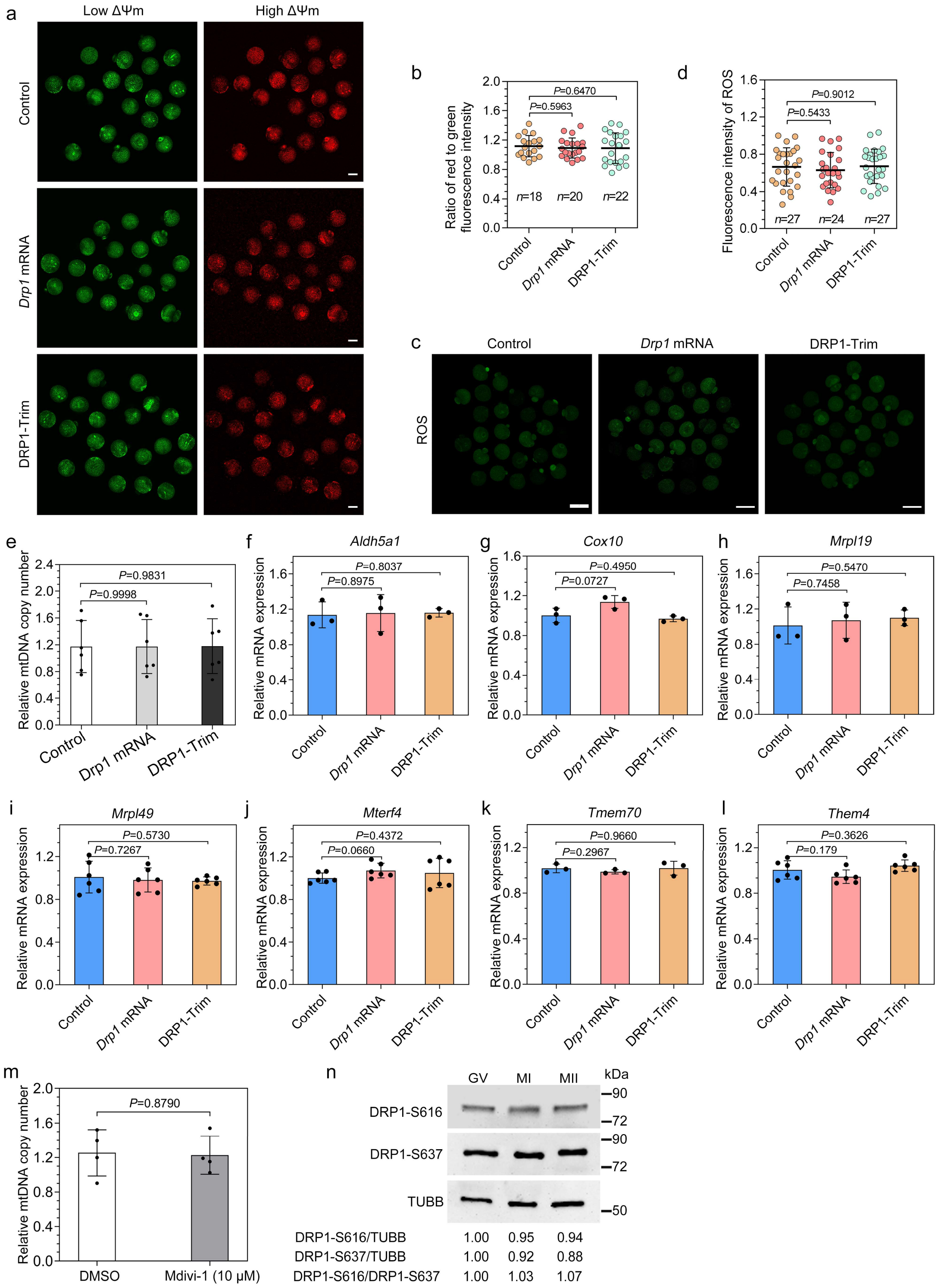

Supplementary Figure 11. *Drp1* overexpression or acute loss has no detectable effects on mitochondrial function during GV to MII transition in oocytes.

a, b Mitochondrial membrane potentials after *Drp1* overexpression or knockdown. Green, low ΔΨm; Red, high ΔΨm. The ratio of red to green fluorescence intensities shows the membrane potentials. Scale

bar, 50 µm.

c, d Fluorescence intensity of ROS (green) was detected after *Drp1* overexpression or knockdown. Scale bar, 100 µm.

e mtDNA copy number was measured after *Drp1* overexpression or knockdown in MII stage oocytes by quantifying the unique mitochondrial sequence with RT-qPCR. Beta-2M was used as the internal control. Six independent replicates were performed.

f-l Relative expression levels of mitochondria-related genes *Aldh5a1, Cox10, Mrpl19, Mrpl49, Mterf4, Tmem70,* or *Them4* in MII stage oocytes after *Drp1* overexpression or knockdown. GV stage oocytes were injected with *Drp1* mRNA or subjected to Trim-Away, then matured to the MII stage. The MII stage oocytes were collected for RT-qPCR. At least three independent replicates were performed for each gene.

m mtDNA copy number in MII stage oocytes was measured after inhibiting DRP1 with Mdivi-1 by quantifying the unique mitochondrial sequence with RT-qPCR. Beta-2M was used as the internal control. Oocytes treated with DMSO were used as the negative control. Four independent replicates were performed.

n Western blot shows DRP1-S616/S637 protein levels during oocyte maturation. Per lysate containing 100 oocytes was probed with anti-DRP1-S616 and anti-DRP1-S637, respectively. β-Tubulin was used as the loading control. One representative blot from three similar independent repeats is shown and quantified with ImageJ. Data are presented as the relative intensity of bands when normalize in GV as 1.00. The data of mean ± standard deviation (S.D.) and *P* values are provided in the Source Data file.

n in graphs refers to the total number of oocytes used for detection. Data are presented as the mean ± standard deviation (S.D.). *P* values were calculated based on unpaired Student's t-test (two-tailed). GV, germinal vesicle; MI, metaphase I; MII, metaphase II. Representative micrographs from at least three independent repeats are shown. Source data are provided as a Source Data file.
