# Supplementary material for: CENP-F-dependent DRP1 function regulates APC/C activity during oocyte meiosis I
Source: Nat Commun. 2022 Dec 13;13:7732. doi: 10.1038/s41467-022-35461-5 (PMC9747930; doi:10.1038/s41467-022-35461-5)
Supplement: Supplementary file 2 — Description of Additional Supplementary Files [file 41467_2022_35461_MOESM2_ESM.docx]

**Description of Additional Supplementary Materials for**

**CENP-F-dependent DRP1 function regulates APC/C activity during oocyte meiosis I**

**File name: Supplementary Movie 1**

**Description:** **Cyclin B1 degradation in TRIM21+IgG injected oocytes, related to Figure 5d.** TRIM21+IgG injected oocyte. Green, eGFP-CyclinB1. Time after NEBD, h:m. A representative movie from at least three independent repeats is shown.

**File name: Supplementary Movie 2**

**Description: Cyclin B1 degradation in TRIM21+anti-DRP1 injected oocytes, related to Figure 5d.** TRIM21+anti-DRP1 injected oocyte. Green, eGFP-CyclinB1. Time after NEBD, h:m. A representative movie from at least three independent repeats is shown.

**File name: Supplementary Movie 3**

**Description: Securin degradation in TRIM21+IgG injected oocytes, related to Figure 5e.** TRIM21+IgG injected oocyte. Red, mCherry-Securin. Time after NEBD, h:m. A representative movie from at least three independent repeats is shown.

**File name: Supplementary Movie 4**

**Description: Securin degradation in TRIM21+anti-DRP1 injected oocytes, related to Figure 5e.** TRIM21+anti-DRP1 injected oocyte. Red, mCherry-Securin. Time after NEBD, h:m. A representative movie from at least three independent repeats is shown.

**File name: Supplementary Movie 5**

**Description: Cyclin B1 degradation in control oocytes, related to Figure 5h.** Control mRNA injected oocyte. Green, eGFP-CyclinB1. Time after NEBD, h:m. A representative movie from at least three independent repeats is shown.

**File name: Supplementary Movie 6**

**Description: Cyclin B1 degradation in *Drp1* mRNA injected oocytes, related to Figure 5h.** *Drp1* mRNA injected oocyte. Green, eGFP-CyclinB1. Time after NEBD, h:m. A representative movie from at least three independent repeats is shown.

**File name: Supplementary Movie 7**

**Description: Securin degradation in control oocytes. Related to Figure 5i.** Control mRNA injected oocyte. Red, mCherry-Securin. Time after NEBD, h:m. A representative movie from at least three independent repeats is shown.

**File name: Supplementary Movie 8**

**Description: Securin degradation in *Drp1* mRNA injected oocytes. Related to Figure 5i.** *Drp1* mRNA injected oocyte. Red, mCherry-Securin. Time after NEBD, h:m. A representative movie from at least three independent repeats is shown.

**File name: Supplementary Movie 9**

**Description: Cyclin B1 degradation of TRIM21+IgG injected oocytes cultured in nocodazole, related to Figure 5l.** TRIM21+IgG injected oocyte. Green, eGFP-CyclinB1. Time after NEBD, h:m. A representative movie from at least three independent repeats is shown.

**File name: Supplementary Movie 10**

**Description: Cyclin B1 degradation of TRIM21+anti-DRP1 injected oocytes cultured in nocodazole, related to Figure 5l.** TRIM21+anti-DRP1 injected oocyte. Green, eGFP-CyclinB1. Time after NEBD, h:m. A representative movie from at least three independent repeats is shown.

**File name: Supplementary Movie 11**

**Description: Securin degradation of TRIM21+IgG injected oocytes cultured in nocodazole, related to Figure 5m.** TRIM21+IgG injected oocyte. Red, mCherry-Securin. Time after NEBD, h:m. A representative movie from at least three independent repeats is shown.

**File name: Supplementary Movie 12**

**Description: Securin degradation of TRIM21+anti-DRP1 injected oocytes cultured in nocodazole, related to Figure 5m.** TRIM21+anti-DRP1 injected oocyte. Red, mCherry-Securin. Time after NEBD, h:m. A representative movie from at least three independent repeats is shown.

**File name: Supplementary Movie 13**

**Description:** **Cyclin B1 degradation in control oocytes, related to Figure 6d.** Control mRNA injected oocyte. Green, eGFP-CyclinB1. Time after NEBD, h:m. A representative movie from at least three independent repeats is shown.

**File name: Supplementary Movie 14**

**Description:** **Cyclin B1 degradation in *Apc2* mRNA injected oocytes, related to Figure 6d.** *Apc2* mRNA injected oocyte. Green, eGFP-CyclinB1. Time after NEBD, h:m. A representative movie from at least three independent repeats is shown.

**File name: Supplementary Movie 15**

**Description:** **Cyclin B1 degradation in *Apc2*+*Drp1* mRNA injected oocytes, related to Figure 6d.** *Apc2* +*Drp1* mRNA injected oocytes. Green, eGFP-CyclinB1. Time after NEBD, h:m. A representative movie from at least three independent repeats is shown.

**File name: Supplementary Movie 16**

**Description:** **Securin degradation in control oocytes, related to Figure 6e.** Control mRNA injected oocyte. Red, mCherry-Securin. Time after NEBD, h:m. A representative movie from at least three independent repeats is shown.

**File name: Supplementary Movie 17**

**Description:** **Securin degradation in *Apc2* mRNA injected oocytes, related to Figure 6e.** *Apc2* mRNA injected oocyte. Red, mCherry-Securin. Time after NEBD, h:m. A representative movie from at least three independent repeats is shown.

**File name: Supplementary Movie 18**

**Description:** **Securin degradation in *Apc2*+*Drp1* mRNA injected oocytes, related to Figure 6e.** *Apc2* +*Drp1* mRNA injected oocytes. Red, mCherry-Securin. Time after NEBD, h:m. A representative movie from at least three independent repeats is shown.
