# Supplementary material for: CENP-F-dependent DRP1 function regulates APC/C activity during oocyte meiosis I
Source: Nat Commun. 2022 Dec 13;13:7732. doi: 10.1038/s41467-022-35461-5 (PMC9747930; doi:10.1038/s41467-022-35461-5)
Supplement: Supplementary file 21 — Reporting Summary [file 41467_2022_35461_MOESM21_ESM.pdf]

nature research

Corresponding author(s): Cheng-Guang Liang, Cheng-Jie Zhou

Last updated by author(s): Nov 29, 2022

Reporting Summary

Nature Research wishes to improve the reproducibility of the work that we publish. This form provides structure for consistency and transparency in reporting. For further information on Nature Research policies, see our Editorial Policies and the Editorial Policy Checklist.

Statistics

For all statistical analyses, confirm that the following items are present in the figure legend, table legend, main text, or Methods section.

n/a	Confirmed	
☐	☒	The exact sample size (*n*) for each experimental group/condition, given as a discrete number and unit of measurement
☐	☒	A statement on whether measurements were taken from distinct samples or whether the same sample was measured repeatedly
☐	☒	The statistical test(s) used AND whether they are one- or two-sided<br>*Only common tests should be described solely by name; describe more complex techniques in the Methods section.*
☒	☐	A description of all covariates tested
☒	☐	A description of any assumptions or corrections, such as tests of normality and adjustment for multiple comparisons
☐	☒	A full description of the statistical parameters including central tendency (e.g. means) or other basic estimates (e.g. regression coefficient) AND variation (e.g. standard deviation) or associated estimates of uncertainty (e.g. confidence intervals)
☐	☒	For null hypothesis testing, the test statistic (e.g. *F*, *t*, *r*) with confidence intervals, effect sizes, degrees of freedom and *P* value noted<br>*Give P values as exact values whenever suitable.*
☒	☐	For Bayesian analysis, information on the choice of priors and Markov chain Monte Carlo settings
☒	☐	For hierarchical and complex designs, identification of the appropriate level for tests and full reporting of outcomes
☒	☐	Estimates of effect sizes (e.g. Cohen's *d*, Pearson's *r*), indicating how they were calculated

Our web collection on statistics for biologists contains articles on many of the points above.

Software and code

Policy information about availability of computer code

Data collection

1. Stained oocytes or chromosome spreads were examined with a Nikon A1R microscope using a 100× oil-immersion objective.
2. Live cell imaging were acquired with Zeiss LSM780 microscope equipped with a 40×objective.
3. Bands on nitrocellulose membranes were detected using an Enhanced Chemiluminescence Detection Kit (Thermo Fisher Scientific) and captured by Tanon 5200.
4. Single oocyte ATP content was determined using Adenosine 5'-triphosphate (ATP) bioluminescent somatic cell assay kit (Sigma-Aldrich), The luminescence intensity was measured using a Luminometer (Pomega GloMax® 20/20, USA).

Data analysis

1. Images from confocal were analyzed with NIS-Element AR3.0 software (Nikon)
2. Cyclin B1 or Securin fluorescence densities in the whole oocyte were measured with Zeiss Blue 2.3 software and exported to the Microsoft Excel 2017.
3. The data were presented as the mean ± S.D. from at least three times. Chromosome and kinetochore morphologies, PB1 extrusion, spindle defects, ATP content per oocyte, mtDNA copy number, mitochondrial membrane potential, ROS and mitochondria related genes expression were analyzed using nonparametric Kruskal-Wallis tests in SPSS 19.0 software (IBM). The inter-sister KT distance, fluorescence intensity of Mad1, Mad2, Bub1and BubR1 were analyzed with unpaired two-tailed Student's t-tests for absolute values and were calculated in Microsoft Excel (2017).
4. For quantification of fluorescence intensity, the data were smoothed with Fit splin/LOWESS analysis in GraphPad Prism 8.0.2.
5. Bands of western blot were quantified with ImageJ (http://rsbweb.nih.gov/ij/).

For manuscripts utilizing custom algorithms or software that are central to the research but not yet described in published literature, software must be made available to editors and reviewers. We strongly encourage code deposition in a community repository (e.g. GitHub). See the Nature Research guidelines for submitting code & software for further information.

Data

Policy information about availability of data

All manuscripts must include a data availability statement. This statement should provide the following information, where applicable:

- Accession codes, unique identifiers, or web links for publicly available datasets
- A list of figures that have associated raw data
- A description of any restrictions on data availability

Source data are provided with this paper. The mass spectrometry proteomics data related to Supplementary Figure 2b-d generated in this study have been deposited into the Zenodo database under accession code 4603598 (https://zenodo.org/record/4603598). The DOI is 10.5281/zenodo.4603598.
Figure 1-6 and Supplementary Figure 1-3, 6-11 are associated with raw data.
All data are available from the corresponding author upon reasonable request.

Field-specific reporting

Please select the one below that is the best fit for your research. If you are not sure, read the appropriate sections before making your selection.

☒ Life sciences ☐ Behavioural & social sciences ☐ Ecological, evolutionary & environmental sciences

For a reference copy of the document with all sections, see nature.com/documents/nr-reporting-summary-flat.pdf

Life sciences study design

All studies must disclose on these points even when the disclosure is negative.

Sample size	For oocyte maturation experiment, oocytes from at least 3 female mice were used for each repeat (PMID:34764261). For chromosome and centromere statistics, at least 10 oocytes (more than 400 kinetochores) were calculated (PMID: 31679939). In general, immunofluorescence, western blots and Co-immunoprecipitations (Co-IP) experiments were repeated a minimum of 3 independent times (detailed n is indicated in the figure or figure legends). All the data were collected to perform statistical analysis.
Data exclusions	No data was excluded from the analysis.
Replication	All the experiments were reliably reproduced and all the replication were successful. The number of independent experiments were specified in the figure legends.
Randomization	Strains and conditions that were directly compared were typically cultured together. Microscopy image acquisition was performed randomly. All samples were allotted randomly into experimental groups. Further randomization was not applicable.
Blinding	Investigators were not blinded. Blinding was technically difficult because experiments and analysis was carried out by the same investigators.

Reporting for specific materials, systems and methods

We require information from authors about some types of materials, experimental systems and methods used in many studies. Here, indicate whether each material, system or method listed is relevant to your study. If you are not sure if a list item applies to your research, read the appropriate section before selecting a response.

Materials & experimental systems

n/a	Involved in the study	
☐	☒	Antibodies
☐	☒	Eukaryotic cell lines
☒	☐	Palaeontology and archaeology
☐	☒	Animals and other organisms
☒	☐	Human research participants
☒	☐	Clinical data
☒	☐	Dual use research of concern

Methods

n/a	Involved in the study	
☒	☐	ChIP-seq
☒	☐	Flow cytometry
☒	☐	MRI-based neuroimaging

Antibodies

Antibodies used	Immunoblotting<br>rabbit anti-CENPF (Abcam ab5; 1:500), mouse anti-DRP1 (Abcam ab56788; 1:200), rabbit anti-DRP1 (Ser616) Antibody (Cell Signaling Technology 3455; 1:400), rabbit anti-DRP1 (Ser637) Antibody (Cell Signaling Technology 4867; 1:400), rabbit anti-beta β-Tubulin (loading Control, Abcam ab6046; 1:500), mouse anti-cMyc (Thermo Fisher R950-25; 1:400), mouse anti-EGFP (Abcam ab184601; 1:400), rabbit anti-Cyclin B1 (Cell Signaling 4138; 1:200), goat anti-Cyclin B2 (R&D Systems AF6004; 1:400), rabbit anti-flag (Sigma-Aldrich F7425; 1:400), rabbit anti-Securin (Cell Signaling 13445; 1:200), rabbit anti-APC2 (Cell Signaling 12301S; 1:200), rabbit anti-

APC11 (Abcam ab154546; 1:200), rabbit anti-UbcH5/Ubc4 (Proteintech 28328-AP; 1:200), and rabbit anti-ubiquitin (PTM BIO PTM-1106; 1:200). Secondary antibodies used were Peroxidase-conjugated secondary Goat Anti-Mouse (Jackson Immuno Research Laboratories 115-035-003; 1:2000), Donkey Anti-Rabbit (Jackson 711-035-152; 1:2000), and Donkey Anti-Goat (Jackson 705-035-003; 1:2000).
Immunofluorescence
rabbit anti-CENPF (Abcam ab5; 1:700), mouse anti-Lamin B1 (Abcam ab8982; 1:400), human anti-centromere (Antibodies Incorporated 15-234-0001; 1:500), mouse anti-DRP1 (Abcam ab56788; 1:200) and, mouse anti-Tubulin (Abcam; 1:1000) mouse rabbit anti-Tubulin (Abcam ab6046; 1:500 for whatAbcam ab44928; 1:1000 for what) and mouse anti-cMyc (Thermo Fisher R950-25; 1:200). Secondary antibodies used were Rhodamine (TRITC) AffiniPure Donkey Anti-Rabbit (Jackson 711-025-152; 1:750), Alexa Fluor 647 AffiniPure Donkey Anti-Human (Jackson 709-605-149; 1:500), and Alexa Fluor 488 AffiniPure Donkey Anti-Mouse (Jackson 715-545-151; 1:500).
Chromosome spreads
rabbit anti-CENPF (Abcam ab5; 1:50), mouse anti-DRP1 (Abcam ab56788; 1:50), rabbit anti-DRP1 (Abcam ab180769; 1:100), mouse anti-DRP1 (BD Biosciences 611112; 1:50), rabbit anti-BUB1 (Abcam ab9000; 1:50), rabbit anti-BUBR1 (Proteintech 115042-AP; 1:100), rabbit anti-APC2 (Proteintech 13559-1-AP; 1:50), rabbit anti-MAD1 (Abcam ab175245; 1:50), rabbit anti-MAD2 (Biolegend 924601; 1:50), human anti-centromere (CREST, Antibodies Incorporated 15-234-0001; 1:100), rabbit anti-REC8 (Proteintech 10793-1-AP; 1:50), rabbit anti-SMCmc3 (Abcam ab128919; 1:50) and mouse anti-Myc (Thermo Fisher R950-25; 1:100). Alexa Fluor 647 AffiniPure Donkey Anti-Human (1:50), Rhodamine (TRITC) AffiniPure Donkey Anti-Mouse (Jackson 715-025-150; 1:100), Alexa Fluor 488 AffiniPure Donkey Anti-Mouse (1:100), Alexa Fluor 488 AffiniPure Donkey Anti-Rabbit (Jackson 711-545-152; 1:100), and Rhodamine (TRITC) AffiniPure Donkey Anti-Rabbit (1:100).

Validation

The validation information of all antibodies used in our study is provided as follows. For the information from the vendor, the hyperlinks are provided; for the information from references, the PMID is provided.
Rabbit polyclonal anti-CENPF (Abcam ab5), raised against the C-terminus of CENP-F (Human), suitable for IF and WB, reacts with Mouse (https://www.abcam.com/cenpf-antibody-ab5.html PMID: 30856164)
Mouse monoclonal anti-DRP1 (Abcam ab56788), raised against full-length protein corresponding to Human DRP1 aa 1-710, suitable for IP, IF and WB, reacts with Mouse, Human (https://www.abcam.com/drp1-antibody-3b5-ab56788.html PMID: 33535046, 27739424)
Rabbit polyclonal anti-DRP1(Ser616) (Cell Signaling Technology 3455), synthetic phosphopeptides corresponding to Ser616 surrounding residues of DRP1 (Human), suitable for WB, reacts with Mouse (https://www.cellsignal.com/products/primary-antibodies/phospho-drp1-ser616-antibody/3455 PMID: 36163170)
Rabbit polyclonal anti-DRP1(Ser637) (Cell Signaling Technology 4867), synthetic phosphopeptides corresponding to Ser637 surrounding residues of DRP1 (Human), suitable for WB, reacts with Mouse (https://www.cellsignal.com/products/primary-antibodies/phospho-drp1-ser637-antibody/4867 PMID: 36263130)
Rabbit polyclonal anti-beta Tubulin (Abcam ab6046), Synthetic peptide, suitable for WB, reacts with Mouse (https://www.abcam.com/beta-tubulin-antibody-loading-control-ab6046.html PMID: 32054943)
Mouse monoclonal anti-cMyc (Thermo Fisher R950-25), c-Myc synthetic peptide: Glu-Gln-Lys-Leu-Ile-Ser-Glu-Glu-Asp-Leu-, suitable for IP, IF and WB, reacts with Mouse, Human (https://www.thermofisher.cn/cn/zh/antibody/product/Myc-Tag-Antibody-Monoclonal/R950-25 PMID: 28660881, 12915472, 32562308)
Mouse monoclonal anti-EGFP (Abcam ab184601), raised against full-length protein corresponding to EGFP aa 1 to the C-terminus, suitable for WB, reacts with Mouse (https://www.abcam.com/egfp-antibody-f56-6a123-ab184601.html PMID: 32589305)
Rabbit polyclonal anti-Cyclin B1 (Cell Signaling Technology 4138), raised against a synthetic peptide corresponding to the residue around the amino terminal of human cyclin B1, suitable for WB, reacts with Mouse (https://www.cellsignal.com/products/primary-antibodies/cyclin-b1-antibody/4138 PMID: 36090903)
Goat polyclonal anti-Cyclin B2 (R&D Systems AF6004), raised against E. coli-derived recombinant mouse Cyclin B2 Ala2-Leu101, suitable for WB, reacts with Mouse (https://www.rndsystems.com/cn/products/human-mouse-cyclin-b2-antibody_af6004 PMID: 34559563)
Rabbit polyclonal anti-Flag (Sigma-Aldrich F7425), raised against peptide sequence DYKDDDDK, suitable for WB, reacts with Mouse (https://www.sigmaaldrich.cn/CN/zh/product/sigma/f7425 PMID: 32444656)
Rabbit monoclonal anti-Securin (Cell Signaling Technology 13445), synthetic peptide corresponding to the residue around Val19 in PTTG1 protein was used, suitable for WB, reacts with Mouse (https://www.cellsignal.com/products/primary-antibodies/securin-d2b6o-rabbit-mab/13445 PMID: 35988650)
Rabbit polyclonal anti-APC2 (Cell Signaling Technology 12301S), synthetic peptides corresponding to residues around Lys458 of human APC2 protein, suitable for IP and WB, reacts with Human, Mouse (https://www.cellsignal.com/products/primary-antibodies/apc2-antibody/12301 PMID: 32905768, 34382737)
Rabbit polyclonal anti-APC11 (Abcam ab154546), synthetic peptide, corresponding to a region within amino acids 26-84 of Apc11 (Human), suitable for WB, reacts with Mouse (https://www.abcam.com/apc11-antibody-ab154546.html)
Rabbit polyclonal anti-UbcH5/Ubc4 (Proteintech 28328-1-AP), raised against UBE2D1/2/3/4 fusion protein Ag28736, suitable for WB, reacts with Mouse (https://www.ptgcn.com/products/UBE2D1-2-3-4-Antibody-28328-1-AP.htm)
Rabbit polyclonal anti-ubiquitin (PTM BIO PTM-1106), raised against a N-terminus of the human ubiquitin, suitable for WB, reacts with Mouse (https://www.ptmbiolabs.com/product/ptm-1106/)
Mouse monoclonal anti-Lamin B1 (Abcam ab8982), raised against a full length native protein (purified) corresponding to Rat Lamin B1 (C terminal), suitable for IF, reacts with Mouse (https://www.abcam.com/lamin-b1-antibody-119d5-f1-nuclear-envelope-marker-ab8982.html PMID: 20658144)
Human polyclonal anti-centromere (Antibodies Incorporated 15-234), Synthetic peptide, suitable for IF, reacts with Mouse (https://www.antibodiesinc.com/products/anti-centromere-protein-antibody-15-234)
Mouse monoclonal anti-Tubulin (Abcam ab44928), raised against a native chick brain microtubules and suitable for IF, reacts with Mouse (https://www.abcam.com/tubulin-antibody-dm1a-dm1b-loading-control-ab44928.html PMID: 32562308)
Rabbit polyclonal anti-DRP1 (Abcam ab180769), raised against a recombinant fragment within Human DRP1 (C terminal), suitable for IF, reacts with Mouse (https://www.abcam.com/drp1-antibody-c-terminal-ab180769.html)
Mouse anti-DRP1 (BD Biosciences 611112), raised against a Rat DLP1 aa. 601-722, suitable for IF, reacts with Mouse (https://www.bdbiosciences.com/en-us/search-results?searchKey=611112 PMID: 17003040)
Rabbit polyclonal anti-BUB1 (Abcam ab9000), raised against a recombinant fragment (His-tag) within Human Bub1 aa 1-350 (N terminal), suitable for IF, reacts with Mouse (https://www.abcam.com/bub1-antibody-ab9000.html PMID: 33248027)
Rabbit polyclonal anti-BUBR1 (Proteintech 115042-AP), raised against a BubR1 fusion protein Ag2053, suitable for IF, reacts with Mouse (https://www.ptgcn.com/products/BUB1B-Antibody-11504-2-AP.htm PMID: 25123474)

Rabbit polyclonal anti-APC2 (Proteintech 13559-1-AP), raised against a APC2 fusion protein Ag4427, suitable for IF, reacts with Mouse (https://www.ptgcn.com/products/ANAPC2-Antibody-13559-1-AP.htm PMID: 32687671)
Rabbit polyclonal anti-MAD1 (Abcam ab175245), raised against a recombinant full length protein corresponding to Human MAD1L1/MAD1 aa 1 to the C-terminus, suitable for IF, reacts with Mouse (https://www.abcam.com/mad1l1mad1-antibody-ab175245.html PMID: 27557495)
Rabbit polyclonal anti-MAD2 (Biolegend 924601), raised against a recombinant full length human MAD2 protein, suitable for IF, reacts with Mouse (https://www.biolegend.com/en-us/products/anti-mad2-antibody-11080 PMID: 31645568)
Rabbit polyclonal anti-REC8 (Proteintech 10793-1-AP), raised against a REC8 fusion protein Ag1250, suitable for IF, reacts with Mouse (https://www.ptgcn.com/products/REC8-Antibody-10793-1-AP.htm PMID: 28590163)
Rabbit monoclonal anti-Smc3 (Abcam ab128919)�synthetic peptide within Human SMC3 aa 1200 to the C-terminus (C terminal), suitable for IF, reacts with Mouse (https://www.abcam.com/smc3-antibody-epr7984-ab128919.html PMID: 31704793)

Eukaryotic cell lines

Policy information about cell lines

Cell line source(s)	The HEK293 cell was from ATCC (CRL-1573)
Authentication	Authentication results can be found at https://www.atcc.org/products/crl-1573
Mycoplasma contamination	The HEK293 cells are tested negative for mycoplasm contamination.
Commonly misidentified lines (See ICLAC register)	There are no misidentified lines in this study.

Animals and other organisms

Policy information about studies involving animals; ARRIVE guidelines recommended for reporting animal research

Laboratory animals	Six-week-old female C57BL/6 and B6D2 F1 mice were purchased from Inner Mongolia University
Wild animals	No wild animals were used in the study
Field-collected samples	All mice were maintained in a specific pathogen-free condition in a controlled environment of 20-22°C, humidity of 40-70% , a 12/12 h light/dark cycle, and with access to chow and water ad libitum. At the end of experiments, mice were euthanized with carbon dioxide followed by cervical dislocation.
Ethics oversight	All studies adhered to procedures consistent with the National Research Council Guide for the Care and Use of Laboratory Animals and were approved by the Institutional Animal Care and Use Committee at Inner Mongolia University.

Note that full information on the approval of the study protocol must also be provided in the manuscript.
