# Supplementary material for: CENP-F-dependent DRP1 function regulates APC/C activity during oocyte meiosis I
Source: Nat Commun. 2022 Dec 13;13:7732. doi: 10.1038/s41467-022-35461-5 (PMC9747930; doi:10.1038/s41467-022-35461-5)

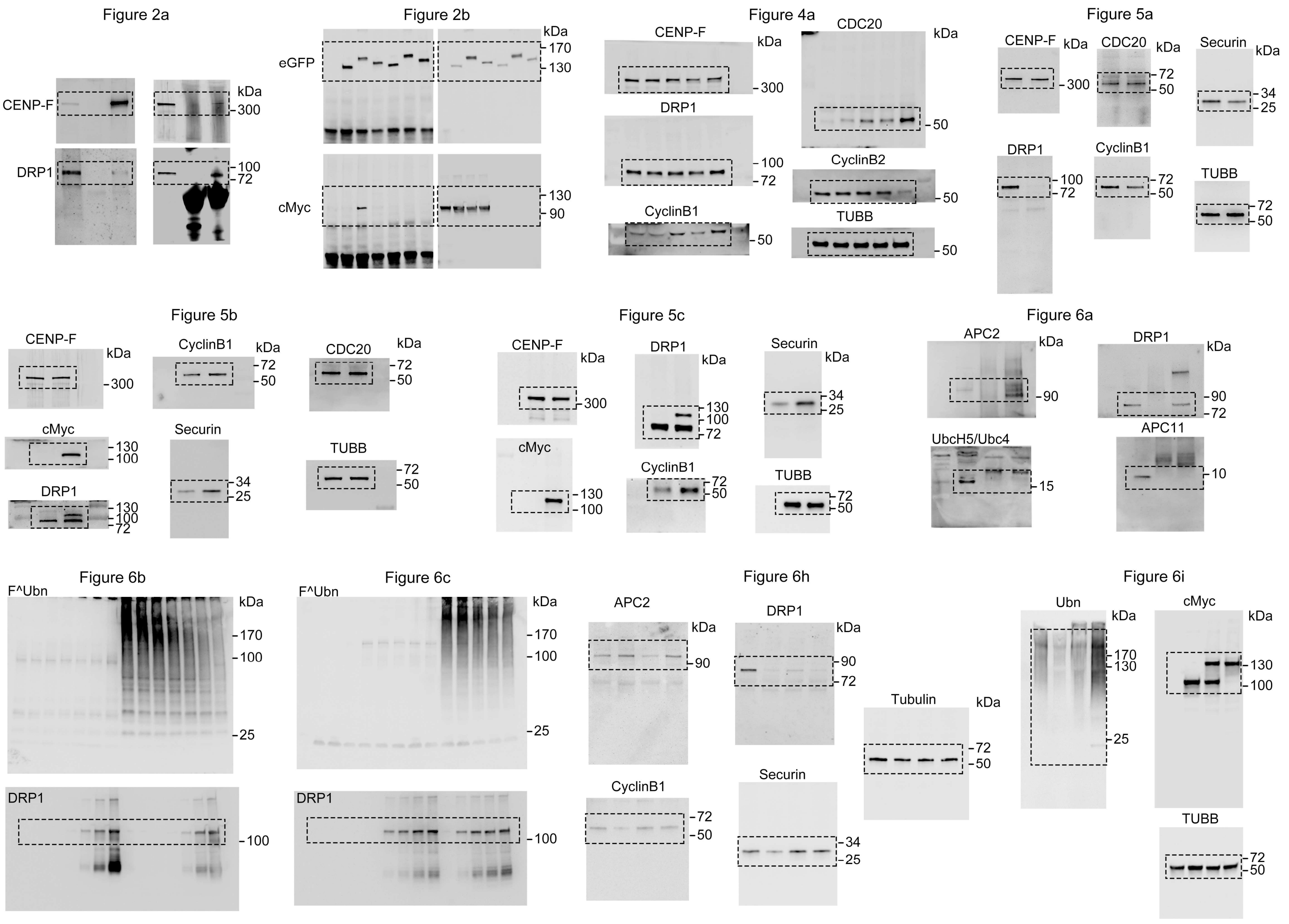
Figure 2a
CENP-F
DRP1
kDa
300
100
72
Figure 2b
eGFP
cMyc
kDa
170
130
90
Figure 4a
CENP-F
DRP1
CyclinB1
CDC20
CyclinB2
TUBB
kDa
300
100
72
50
Figure 5a
CENP-F
CDC20
Securin
DRP1
CyclinB1
TUBB
kDa
300
72
50
34
25
100
Figure 5b
CENP-F
CyclinB1
CDC20
cMyc
Securin
TUBB
DRP1
kDa
300
72
50
130
100
34
25
Figure 5c
CENP-F
DRP1
Securin
cMyc
CyclinB1
TUBB
kDa
300
130
100
72
34
25
50
Figure 6a
APC2
DRP1
UbcH5/Ubc4
APC11
kDa
90
72
15
10
Figure 6b
F^Ubn
DRP1
kDa
170
100
25
Figure 6c
F^Ubn
DRP1
kDa
170
100
25
Figure 6h
APC2
DRP1
Tubulin
CyclinB1
Securin
kDa
90
72
50
34
25
Figure 6i
Ubn
cMyc
TUBB
kDa
170
130
25
100
72
50

Supplementary Figure 1b

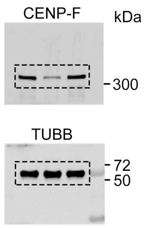

Supplementary Figure 2b

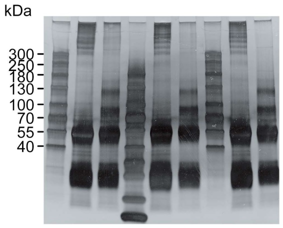

Silver staining gel
IP: anti-CENP-F

Supplementary Figure 3g

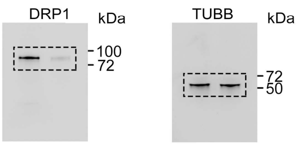

Supplementary Figure 6a

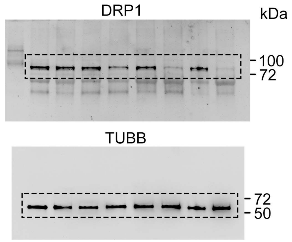

Supplementary Figure 8

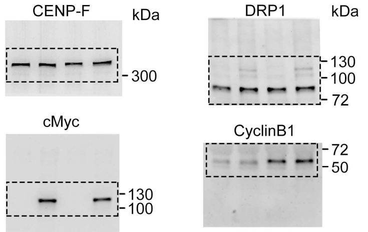

Supplementary Figure 8

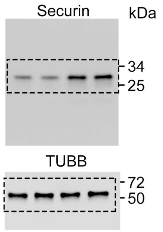

Supplementary Figure 9a

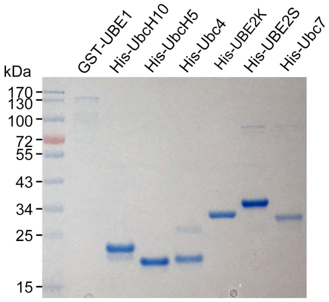

Supplementary Figure 9b

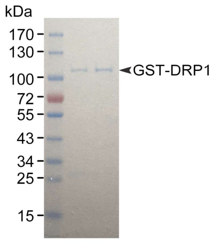

Supplementary Figure 9c

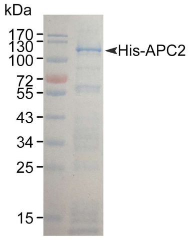

Supplementary Figure 9d

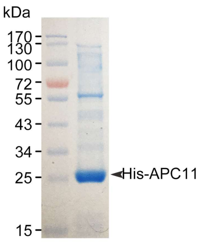

Supplementary Figure 9e

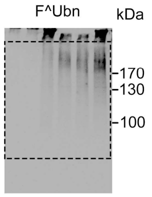

Supplementary Figure 9f

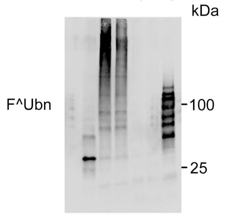

Supplementary Figure 9i

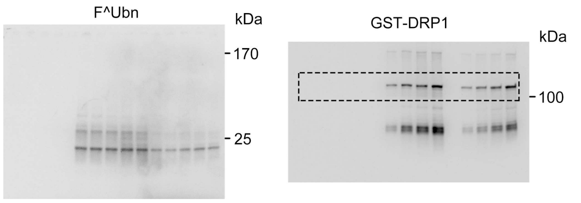

Supplementary Figure 9j

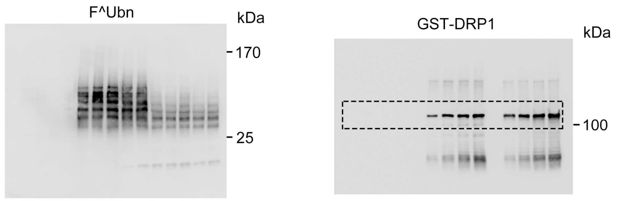

Supplementary Figure 9k

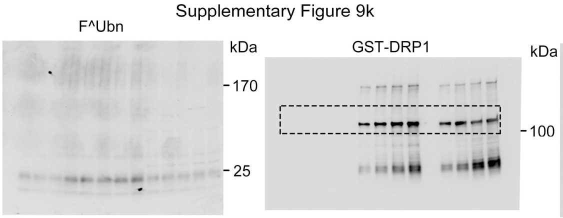

Supplementary Figure 9l

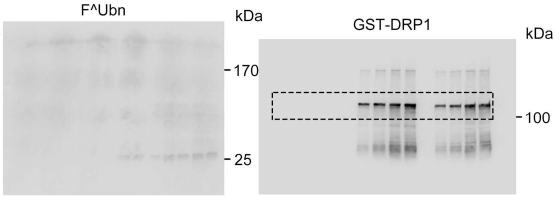

Supplementary Figure 11n

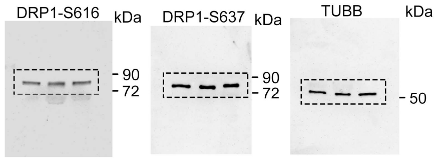

Supplement: Supplementary file 22 — Source Data [file 41467_2022_35461_MOESM22_ESM.zip › Source Data 2 uncropped WB.pdf]
